# Advances in Polydopamine-Based Nanoplatforms: Antioxidant Mechanisms and Applications in Oxidative Stress-Mediated Diseases

**DOI:** 10.1007/s40820-026-02205-9

**Published:** 2026-05-18

**Authors:** Zhilin Wang, Zhu Liu, Luming Song, Xinyi Zhao, Shuaipeng Feng, Donghua Di, Hao Ju, Long Wan, Qinfu Zhao

**Affiliations:** 1 Department of Pharmaceutics, School of Pharmacy, Shenyang Pharmaceutical University, Shenyang, 110016 People’s Republic of China; 2 Department of Microbial and Biochemical Pharmacy, School of Life Sciences and Biopharmaceuticals, Shenyang Pharmaceutical University, Shenyang, 110016 People’s Republic of China; 3 Department of Ultrasound, Shengjing Hospital of China Medical University, Shenyang, 110004 People’s Republic of China; 4 Department of Pharmacy, The First Hospital of China Medical University, 155 Nanjing North Street, Shenyang, 110001 People’s Republic of China; 5 Joint International Research Laboratory of Intelligent Drug Delivery Systems, Ministry of Education, Shenyang, 110016 People’s Republic of China

**Keywords:** Polydopamine-based nanoplatforms, Physicochemical properties, Antioxidant nanozyme, Reactive oxygen species, Oxidative stress-mediated diseases

## Abstract

Polydopamine (PDA) exhibits unique advantages in the treatment of oxidative damage owing to its melanin-mimetic structure, abundant redox-active functional groups, and excellent biocompatibility. Distinct from conventional antioxidant molecules, PDA-based nanoplatforms can efficiently eliminate reactive oxygen species (ROS) via hydrogen atom transfer and electron transfer mechanisms, while relying on the dynamic redox cycling of catechol/quinone moieties to achieve sustained antioxidant activity. However, systematic summaries of PDA-based antioxidant nanoplatforms remain relatively limited. Therefore, this review provides a comprehensive overview of the antioxidant mechanisms of PDA and its associated physicochemical properties, with particular emphasis on the design strategies of diverse PDA-based nanoplatforms, including solid, mesoporous, hollow, doped, and coated architectures, as well as the effects of structural features, particle size, composition, and surface charge on their antioxidant performance. In addition, recent research progress is systematically categorized around core pathological challenges, including breaking ROS-inflammation feedback loops, overcoming biological delivery barriers, remodeling regenerative microenvironments, and regulating programmed cell death.

## Highlights

- The antioxidant mechanisms and physicochemical properties of polydopamine (PDA) have been systematically outlined, with emphasis on the dynamic redox cycling of catechol/quinone moieties and strategies for modulating its antioxidant performance.
- The diverse applications of PDA based nanoplatforms in oxidative stress mediated diseases are systematically reorganized according to pathological challenges and microenvironmental features.
- The clinical translation challenges and future development perspectives of PDA based nano platforms have been discussed and proposed in depth, covering pharmacokinetics, long term biosafety, manufacturing feasibility, and emerging directions such as single-atom engineering, multi-omics analysis, and AI-assisted rational design.

## Graphical abstract

**Figure:**
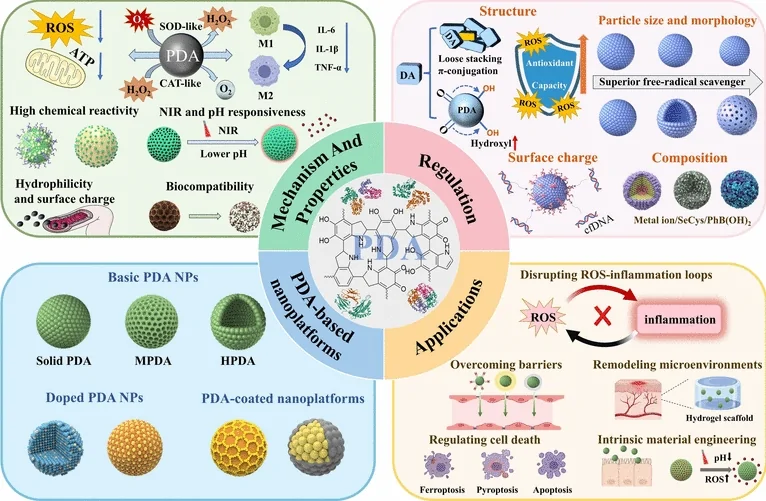

## Introduction

Redox reactions are indispensable throughout the life processes of living organisms. Oxygen, a key participant in redox reactions, is abundant in biological systems and readily accepts free electrons generated during normal cellular oxidative metabolism, leading to the generation of various molecular oxygen derivatives, commonly referred to as reactive oxygen species (ROS), such as superoxide anion (O_2_·^−^), hydroxyl radicals (·OH), and hydrogen peroxide (H_2_O_2_) [1, 2]. At physiological levels, ROS play essential roles in regulating cellular homeostasis and serve as important signaling mediators involved in the pathophysiological processes of various diseases. In contrast, when redox homeostasis is disrupted, excessive ROS accumulation triggers oxidative stress-driven molecular damage, impairing nucleic acids, membrane lipids, and proteins and thereby accelerating inflammatory and degenerative pathological processes [3–5]. Therefore, the preservation of redox homeostasis is crucial for preventing oxidative stress-induced damage. To regulate ROS levels, living organisms have evolved a complex three-tier antioxidant defense system [6]. The first-line defense comprises endogenous antioxidant enzymes, such as superoxide dismutase (SOD), catalase (CAT), and glutathione peroxidase (GPx) [7]. The second-line defense involves exogenous dietary-derived small-molecule antioxidants, including vitamin C, vitamin E, and carotenoids [8]. The third-line defense consists of enzyme-mediated systems responsible for repairing and removing oxidized proteins and other biomolecules. However, natural antioxidant enzymes exhibit several limitations in practical applications, including poor stability, high cost, environmental sensitivity of catalytic activity, and susceptibility to inactivation under excessive ROS conditions [9]. Consequently, antioxidant nanozymes with high stability and facile preparation have been explored as alternatives to natural enzymes [10]. Nevertheless, nanozymes generally display lower selectivity and catalytic activity than their natural counterparts and often suffer from poor biocompatibility, which severely restricts their clinical translation. Therefore, the development of biomaterials with high biocompatibility and potent antioxidant activity is highly desirable.

Polydopamine (PDA) is a biomimetic polymer inspired by mussel adhesive proteins and has garnered considerable attention in the fields of biomaterials and biomedicine owing to its excellent biocompatibility, strong surface adhesion capability, and abundant active functional groups [11]. The synthesis of PDA is straightforward and scalable, typically achieved via solution oxidation using dopamine as a precursor. Under mildly alkaline conditions, dissolved oxygen acts as the oxidant, thereby enabling the spontaneous polymerization of dopamine through deprotonation and oxidation reactions. This process can be conducted under mild conditions without the need for sophisticated equipment and exhibits high controllability and reproducibility [12]. Structurally, PDA features a unique chemical composition rich in active functional groups, such as amine and catechol moieties [13]. These groups not only confer PDA with robust adhesion, allowing stable coating on virtually all material surfaces—including metals, ceramics, and diverse polymers—but also make PDA an ideal platform for surface modification. Consequently, PDA has been extensively applied in the functionalization of tissue engineering scaffolds, implantable devices, and drug delivery systems, thereby facilitating the development of tailored therapeutic platforms for oxidative stress-mediated diseases. In addition, PDA exhibits excellent biosafety profiles in biomedical applications, demonstrating favorable tissue compatibility and reduced immune rejection, which supports its long-term in vivo use [14]. Notably, PDA possesses intrinsic antioxidant activity, as catechol groups within its molecular structure can directly capture and neutralize ROS through reversible redox reactions [15], thereby alleviating oxidative stress. While natural antioxidant enzymes exhibit high catalytic efficiency, they suffer from inherent protein fragility leading to poor environmental stability and high production costs. In stark contrast, PDA functions as a robust antioxidant nanozyme that overcomes these biological limitations by offering superior long-term stability and maintaining its functional integrity under complex pathological conditions. Furthermore, the exceptional structural tunability of PDA alongside its multi-mechanistic synergistic effects ranging from concurrent hydrogen atom transfer and electron transfer to potent metal-ion chelation establishes it as a unique platform capable of mimicking the cooperative action of multiple natural enzymes. Accordingly, PDA has demonstrated promising therapeutic potential in various pathological models, including inflammation-associated diseases, neurodegenerative disorders, and ischemia–reperfusion injury. Collectively, these multifunctional characteristics endow PDA with broad application prospects in antioxidant therapy and intervention strategies for oxidative stress-mediated diseases, continuously driving innovation and advancement in biomimetic biomaterials.

**Scheme 1:**
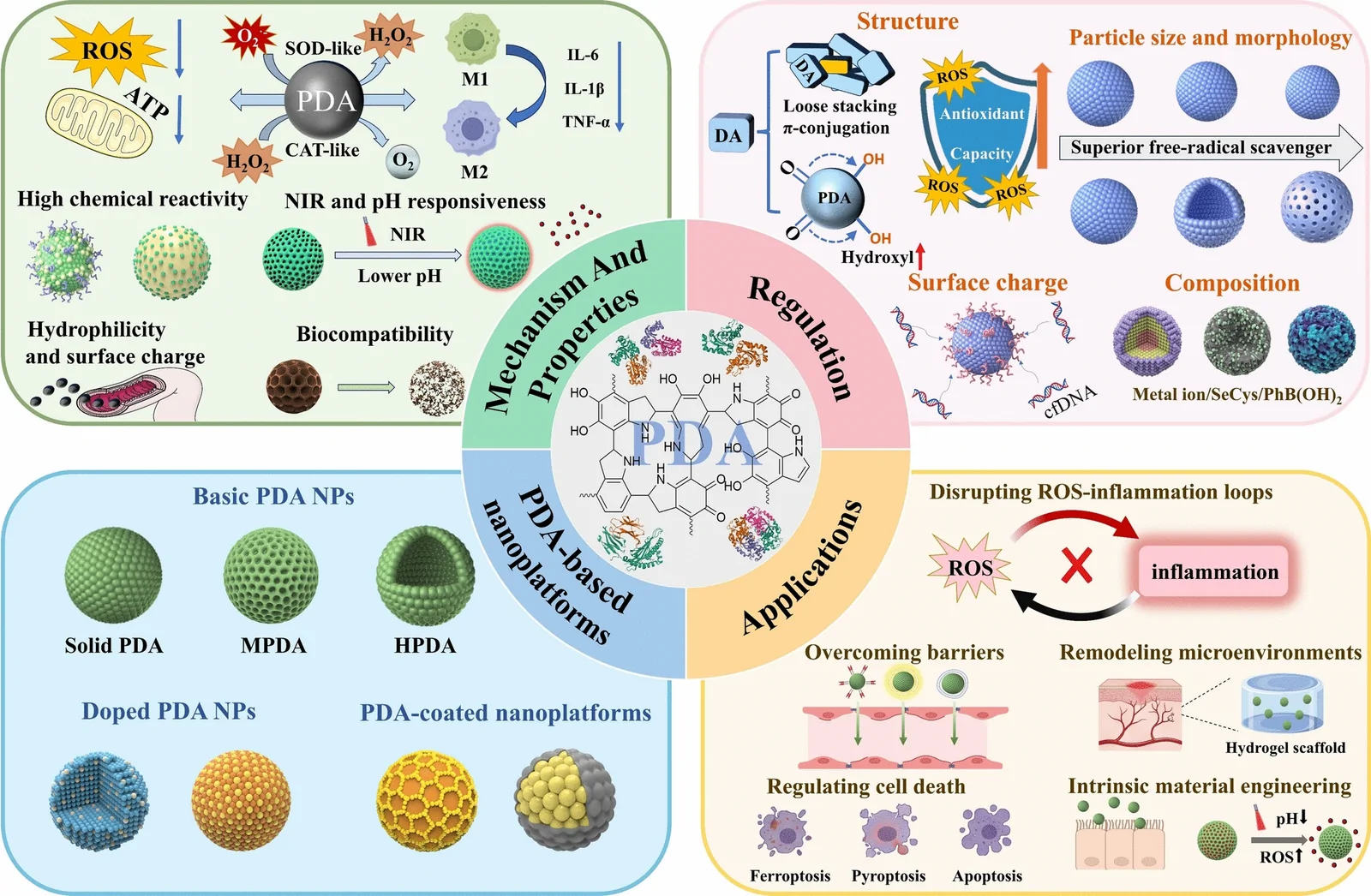
The properties, regulation, classification, and applications of PDA-based nanoplatforms

Although a growing body of studies has explored the biomedical applications of nano-antioxidants [16–18], and several reviews addressing recent advances in PDA research have been published [19–21], a systematic and comprehensive overview dedicated to PDA-based nanoplatforms for the treatment of oxidative stress-mediated diseases remains lacking. PDA not only exhibits excellent ROS-scavenging capability and diverse enzyme-mimicking activities but also possesses favorable biocompatibility, tunable physicochemical properties, and abundant surface functional groups, enabling versatile applications in drug delivery, inflammation modulation, and tissue repair. Based on the physicochemical characteristics and antioxidant mechanisms of PDA, this review systematically discusses its structural optimization strategies, construction approaches for drug delivery systems, and therapeutic applications organized by design strategies for addressing specific pathological hurdles, such as immune modulation, barrier penetration, and cell death regulation (Scheme 1). By providing an in-depth analysis of the antioxidant properties of PDA and a comprehensive overview of its applications in oxidative stress-mediated diseases, this review aims to offer new insights into the rational design of PDA-based nanoplatforms, facilitate their translation from fundamental research to clinical therapy, and provide theoretical foundations and perspectives for the future development of antioxidant nanomaterials.

## Antioxidant Mechanisms and Physicochemical Properties

PDA, a biomimetic polymer inspired by mussel adhesive proteins [22], has emerged as a highly promising candidate in biomedical applications, particularly for interventions targeting oxidative stress-mediated diseases [23], attributable to its unique physicochemical properties and exceptional antioxidant capability. Accordingly, a comprehensive understanding of the intrinsic relationship between the physicochemical characteristics and antioxidant mechanisms of PDA is essential to the rational design and translational application of PDA-based nanoplatforms for the treatment of oxidative stress-mediated diseases.

### Antioxidant Mechanisms

**Scheme 2:**
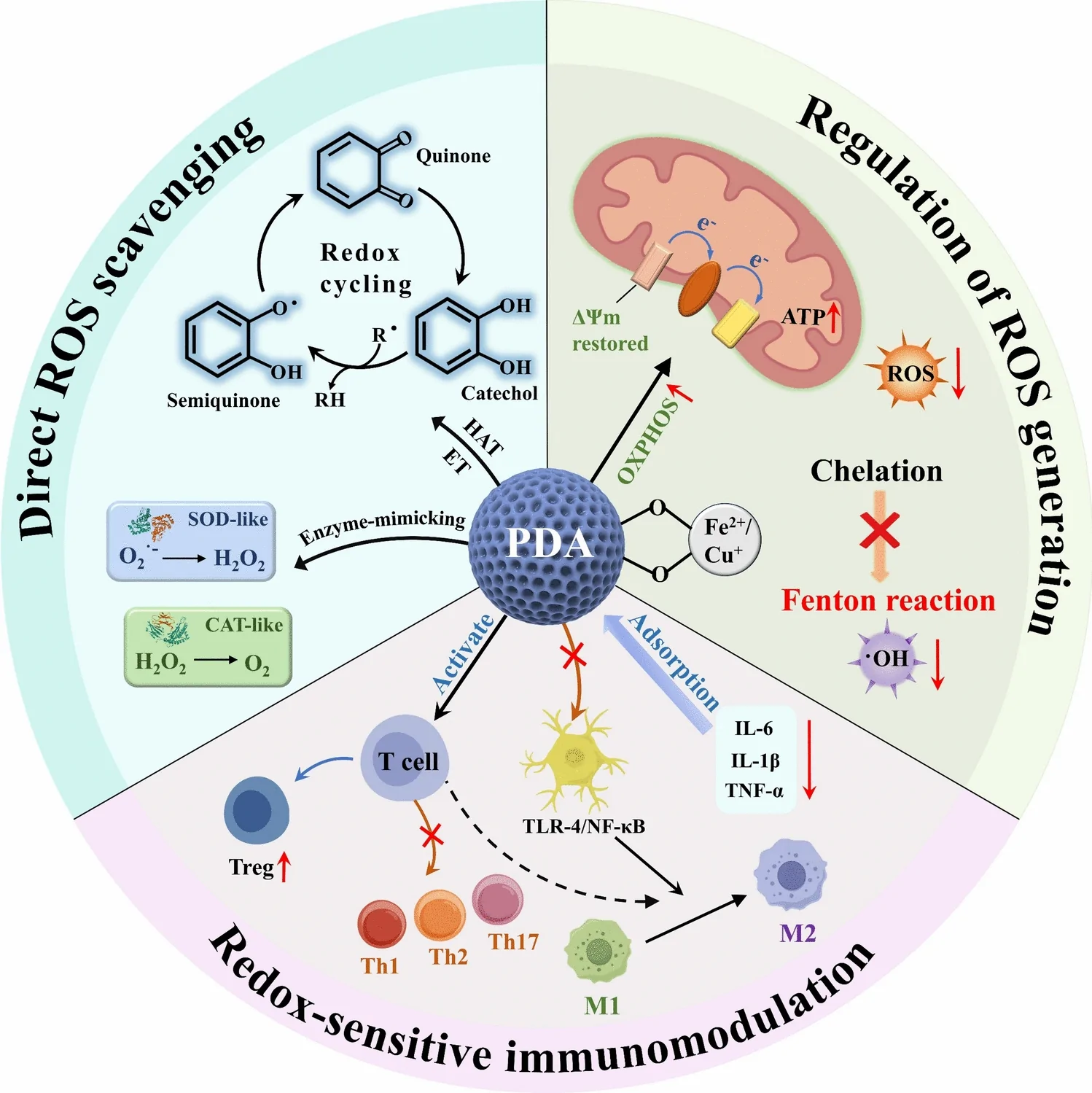
Antioxidant mechanisms of PDA. Schematic illustration of the three primary pathways: (1) direct ROS scavenging, (2) regulation of ROS generation, and (3) redox-sensitive immunomodulation

The antioxidant mechanism of PDA is characterized by its distinct comprehensiveness. On the one hand, the abundant catechol and quinone units within its structure can directly participate in radical-scavenging reactions to thereby reduce ROS concentrations at the molecular level. On the other hand, PDA can indirectly influence the generation and accumulation of ROS through pathways such as the regulation of mitochondrial function and the inhibition of metal-ion-catalyzed processes. Furthermore, fluctuations in oxidative stress levels often trigger responses from a series of inflammation-related signaling pathways where the modulation of ROS by PDA may further influence immune responses and the expression of inflammatory cytokines. Based on these perspectives, this section will provide a systematic analysis covering three primary aspects including direct scavenging mechanisms, the regulation of ROS generation, and immunomodulation (Scheme 2).

#### Direct ROS Scavenging

As a biomimetic analogue of melanin, PDA is enriched with redox-active structural motifs, and its antioxidant activity does not originate from a single static functional group but rather relies on a dynamic redox network established through the reversible interconversion between catechol and ortho-quinone moieties [24]. Guo et al. [25] systematically elucidated the radical-scavenging mechanism of PDA by combining kinetic analyses with model compound studies, demonstrating that under physiologically relevant peroxyl radical conditions, ortho-quinone units in PDA can be reduced to semiquinone or catechol species via hydrogen atom transfer (HAT) from hydroperoxyl radicals (HO_2_·), thereby initiating an efficient chain-breaking antioxidant process. The semiquinone radicals generated during this reaction process are not transient deactivated species but can achieve electron delocalization within the highly conjugated polymeric network of PDA to thereby attain thermodynamic stability. Semiquinones can either accept further electrons to be reduced to catechols or be re-oxidized into ortho-quinones to form a reversible catechol-semiquinone–ortho-quinone conversion cycle. This cycle enables PDA to maintain a dynamic redox equilibrium during the reaction process rather than acting as a single-use antioxidant. Notably, although catechol groups are traditionally regarded as the primary antioxidant motifs, this study revealed that, within the PDA framework, oxidized ortho-quinone species exhibit higher reactivity under specific radical microenvironments and are capable of sustaining the overall antioxidant capacity through continuous redox cycling. This structural feature of coexisting multiple oxidation states allows PDA to exhibit melanin-like redox buffering behavior and adaptively regulate its electron donor and acceptor functions under varying ROS concentrations.

Beyond the HAT pathway, the highly conjugated structure of PDA and the presence of semiquinone intermediates enable PDA to act as an efficient electron-transfer mediator, allowing single-electron-transfer (ET) processes to participate in radical reduction. Cyclic voltammetry studies have confirmed that PDA possesses a reversible redox potential within the physiological range, enabling it to both accept electrons from reducing agents such as ascorbic acid and donate electrons to oxidizing species to thereby achieve the repeated utilization of its redox activity. This property has been further confirmed by Liu et al. [26] through electrochemical reverse-engineering approaches. Collectively, PDA eliminates ROS primarily through the synergistic operation of HAT and ET mechanisms, and its ROS-scavenging efficiency is governed by both the kinetics of electron transfer and the accessible density of redox-active functional moieties. This dynamic cycle based on the reversible interconversion of catechol and ortho-quinone provides a critical theoretical foundation for the subsequent structural modulation of PDA toward enhanced antioxidant performance.

Meanwhile, the enzyme-mimicking activity of PDA further contributes to its efficient ROS-scavenging capability. Specifically, PDA exhibits SOD-like activity, enabling the rapid elimination of O_2_·^−^ via protonation of pyrrolic nitrogen atoms [27] and catalyzing their disproportionation reaction (2O_2_·^−^ + 2H⁺ → H_2_O_2_ + O_2_). Thereafter, the generated H_2_O_2_ can be decomposed into H_2_O and O_2_ through the CAT-like activity of PDA, thereby preventing H_2_O_2_ from undergoing subsequent Fenton reactions [28]. In addition to these enzyme-mimicking pathways, PDA nanoparticles, as nanomaterials, have been suggested to partially reduce ROS levels in diseased tissues through physical adsorption. Although the specific mechanisms and relative contribution of this adsorption process remain to be fully elucidated, it represents a complementary pathway for ROS elimination by PDA [29]. Notably, previous studies have demonstrated that, among various ROS-scavenging nanoparticles, PDA nanoparticles exhibit markedly enhanced reactivity toward multiple ROS species [30], further underscoring their superior ROS-scavenging performance.

#### Regulation of ROS Generation

Although direct ROS scavenging forms the foundation of the antioxidant effects of PDA, relying solely on the transient neutralization of ROS is often insufficient to maintain long-term homeostasis in complex biological systems. Therefore, an increasing number of studies have focused on whether PDA can reduce oxidative stress levels at the source by regulating the origins of ROS generation. For instance, the improvement of mitochondrial function or the inhibition of metal-ion-catalyzed processes can indirectly reduce the continuous generation of ROS. Consequently, the antioxidant efficacy of PDA is manifested not only at the level of terminal clearance but also involves upstream regulatory mechanisms.

This upstream regulation is exemplified by the ability of PDA to target mitochondrial functional homeostasis, an effect that has been demonstrated in inflammatory disease models. Mitochondria represent the primary site of oxidative stress, generating adenosine triphosphate through oxidative phosphorylation (OXPHOS), while ROS are predominantly produced as by-products of this process. Under oxidative stress conditions, mitochondrial electron transport chain (ETC) dysfunction leads to enhanced electron leakage, resulting in excessive ROS accumulation [31, 32]. PDA is capable of disrupting the vicious cycle between mitochondrial damage and ROS overproduction by preserving mitochondrial structural integrity and optimizing metabolic function. Specifically, PDA restores the activity of key ETC complexes, thereby re-establishing OXPHOS homeostasis and mitochondrial membrane potential. This upstream regulation reduces electron leakage at the source, suppresses excessive ROS generation, and ultimately alleviates oxidative stress-associated pathological manifestations [33]. Furthermore, the abundant active sites of PDA endow it with the capability to further chelate various metal ions, meaning that PDA can effectively sequester overexpressed free metal ions such as Fe^2^⁺ and Cu⁺ to thereby inhibit the Fenton reaction and prevent the generation of harmful substances such as hydroxyl radicals at the source [34].

#### Redox-Sensitive Immunomodulation

When ROS levels fluctuate, multiple intracellular signaling pathways associated with inflammation and immunomodulation are also affected, and the activation states of pathways such as NF-κB and Nrf2 are often closely correlated with oxidative stress. Therefore, the regulation of ROS by PDA may further alter the expression profiles of inflammatory cytokines and the functions of immune cells. This downstream biological response triggered by oxidative stress modulation extends its antioxidant efficacy from mere chemical reactions to the level of immunomodulation. For instance, Li et al. [35] demonstrated that PDA induces the expansion of regulatory T cells, which counteract pro-inflammatory signaling through the secretion of anti-inflammatory cytokines, including IL-10 and TGF-β, thereby suppressing Th1, Th2, and Th17 cell responses. Concurrently, PDA inhibits dendritic cell activation and increases the Treg/Th17 ratio, ultimately attenuating inflammatory responses in pathological tissues. Moreover, PDA suppresses the pro-inflammatory polarization of microglia by modulating the TLR-4/NF-κB signaling pathway, partially reversing abnormal microglial morphology and M1 polarization while promoting macrophage polarization toward the anti-inflammatory M2 phenotype [36]. As a consequence, the release of pro-inflammatory cytokines, including IL-6, IL-1β, and TNF-α, is suppressed, whereas anti-inflammatory cytokines such as IL-10 are upregulated, thereby indirectly contributing to the alleviation of oxidative stress [37–39]. Furthermore, the inherent physicochemical properties of PDA also endow it with unique immunomodulatory pathways. Jiang et al*.* [40] discovered that in a spinal cord injury (SCI) model, PDA nanoparticles can utilize their surface adhesiveness to capture excessive pro-inflammatory cytokines in the cerebrospinal fluid across a broad spectrum through physical adsorption to thereby inhibit the activation of M1 microglia and A1 astrocytes, protect neurons, and promote motor function recovery. This indicates that the immunomodulatory effects of PDA rely not only on signaling pathway regulation but are also closely related to its characteristics as a broad-spectrum cytokine scavenger.

### Physicochemical Properties

The physicochemical properties of PDA largely dictate the functional efficiency and biological adaptability of its antioxidant effects. Unlike traditional small-molecule antioxidants, PDA acts as a nanoscale material where its chemical structure, surface properties, and environmental responsive behaviors collectively influence its interactions with ROS and the cellular microenvironment. This section will analyze aspects including high chemical reactivity, stimuli-responsive characteristics, interfacial behavior, and biocompatibility to reveal the intrinsic correlation between its material attributes and antioxidant performance.

#### High Chemical Reactivity

The high chemical reactivity of PDA plays multifaceted roles in the treatment of oxidative stress-mediated diseases. PDA molecules contain a high density of reactive functional groups, including o-quinone, amine, and catechol moieties [41], which can interact with diverse surfaces or molecules through hydrogen bonding, electrostatic interactions, and π–π stacking. These interactions allow PDA to readily conjugate with drugs, proteins, and other biomolecules [20, 42], representing a core strategy for expanding its applications in oxidative stress-mediated diseases and providing a foundation for the design of structurally diverse PDA-based nanoplatforms.

Despite extensive investigation, the molecular mechanism underlying PDA adhesion has not yet been fully elucidated. Earlier studies generally regarded catechol groups as the dominant contributors to adhesion, as catechols can form hydrogen bonds with hydroxyl groups on material surfaces, coordinate with metal ions, or generate crosslinked networks through oxidative polymerization, thereby enabling strong surface attachment [43, 44]. However, a study by Li et al. [45] demonstrated that PDA adhesion is not solely dependent on catechol moieties. The aminoethyl side chain of the dopamine precursor was shown to play a critical role by forming weak interactions with material surfaces via van der Waals forces, thereby assisting the oriented adsorption of catechol groups. To further substantiate the contribution of amine groups to adhesion, Lim et al. [46] reported that polycatechol lacking amine functionalities formed coatings only on metal oxide and hydrophilic surfaces, whereas amine-containing PDA derivatives exhibited distinctive surface-independent adhesion behavior. Collectively, these findings suggest that PDA adhesion arises from the synergistic interplay between the strong interactions mediated by catechol groups and the auxiliary contributions of amine functionalities. Elucidation of this mechanism provides a more precise design rationale for surface functionalization and structural engineering of PDA-based nanoplatforms tailored to different oxidative stress-mediated diseases.

#### NIR and pH Responsiveness

The near-infrared (NIR) and pH responsiveness of PDA represent key features enabling its adaptation to oxidative stress-associated pathological microenvironments and the enhancement of antioxidant therapeutic efficacy. The conjugated aromatic structures within PDA molecules efficiently absorb NIR light and convert photon energy into heat via non-radiative transitions, resulting in localized temperature elevation. This process extends beyond conventional photothermal therapy, as it enhances antioxidant effects through multiple mechanisms and allows on-demand activation via external light control [47, 48]. For example, Zhang et al. [49] successfully developed NIR-responsive microneedles integrated with PDA nanozymes for the treatment of atopic dermatitis. Owing to the photothermal conversion capability of PDA, these microneedles enhanced local microcirculation and inhibited bacterial growth, thereby alleviating inflammation. Moreover, the NIR-induced photothermal effect can trigger structural changes in PDA, enabling PDA-based nanoparticles to function as NIR-responsive photothermal drug-release systems for the precise and controllable release of antioxidant therapeutics. In a cartilage repair study, Luo et al. [50] loaded the chondrogenic inducer kartogenin (KGN) into mesoporous PDA and achieved NIR-triggered photothermal release by exploiting the NIR responsiveness of PDA. Notably, PDA maintains stable photothermal performance and structural integrity under repeated NIR irradiation [51], confirming its excellent photothermal stability. This property supports the feasibility of PDA as a reusable NIR-responsive material for long-term applications and establishes a foundation for its use in diseases requiring repeated photothermal intervention, such as chronic inflammation.

The pH responsiveness of PDA originates from the acid–base ionization of catechol and amine groups along its molecular chains, which dynamically remodels intermolecular hydrogen-bonding networks and π–π stacking, thereby enabling on-demand drug release within inflammatory microenvironments. Under acidic conditions, amine groups undergo protonation (-NH_2_ → -NH₃⁺), while catechol deprotonation is suppressed, resulting in weakened intermolecular hydrogen bonding and π–π stacking interactions. This loosens the PDA framework and facilitates drug release. In contrast, under neutral conditions, extensive catechol deprotonation (-OH → -O^−^) enhances intermolecular electrostatic interactions, leading to a more compact structure that restricts drug release. This behavior significantly reduces drug exposure in normal tissues and minimizes potential side effects [52]. For example, PDA capsules developed by Shirjandi et al. [53] exhibited the fastest drug release at pH 5.5, mimicking inflammatory conditions, whereas the slowest release was observed at pH 7.4. These results demonstrate that PDA undergoes disassembly preferentially within inflammatory lesion sites while remaining structurally stable in normal tissues, thereby providing critical evidence for lesion-targeted drug release in PDA-based delivery systems.

#### Hydrophilicity and Surface Charge

Owing to its abundant polar functional groups, including amine and phenolic hydroxyl moieties, PDA exhibits excellent hydrophilicity and tunable surface charge properties, which collectively constitute the functional basis for its therapeutic performance in oxidative stress-mediated diseases. The polar groups within the PDA backbone form stable hydrogen bonds with water molecules, thereby establishing a hydration layer on the particle surface and enhancing overall hydrophilicity [54]. This hydration layer improves interfacial wettability and stability in biological environments and effectively prolongs drug retention at pathological sites. For example, melatonin-loaded PDA-boronophenylalanine (PBA) nanoparticle eye drops (PPP@MT) prolonged ocular surface retention via a hydrophilic coating, thereby enhancing melatonin bioavailability and efficiently scavenging corneal ROS [55]. Moreover, the hydrophilic mesoporous architecture of PDA enables efficient loading of hydrophobic antioxidant agents [56]. Surface charge regulation represents another core feature of PDA. At high pH values, catechol groups undergo deprotonation, resulting in a negatively charged surface, whereas at low pH, amine groups are protonated, conferring a positive surface charge. This charge-switchable behavior endows PDA with targeting capability in oxidative stress-mediated diseases. For instance, mesoporous PDA@CeO_2_ nanoparticles acquire a positive charge in synovial environments, enabling preferential accumulation in M1 macrophages, followed by NIR laser-triggered release of CeO_2_ to catalytically eliminate ROS and decompose H_2_O_2_ for oxygen generation [51]. Notably, surface charge is directly correlated with the antioxidant activity of PDA. Positively charged PDA surfaces electrostatically attract negatively charged ROS, thereby increasing the probability of ROS interaction with active sites within PDA, which enhances SOD-like activity and overall radical-scavenging efficiency [57].

#### Biocompatibility

Biocompatibility is a fundamental prerequisite for the application of PDA-based nanomaterials as antioxidants in the treatment of oxidative stress-mediated diseases, as excellent biocompatibility not only prevents material-induced secondary tissue damage but also ensures stable in vivo retention, enabling sustained antioxidant functions such as ROS scavenging. Moreover, it provides a safe carrier platform for integration with other antioxidant components. The biocompatibility of PDA is closely associated with its melanin-like molecular structure, which renders PDA essentially non-toxic to cells and tissues [13]. For example, Zhou et al. [58] demonstrated in a postoperative adhesion prevention model that although basic MnO_2_ exhibits ROS-scavenging antioxidant activity, it possesses poor biocompatibility and readily induces hemolysis, along with pronounced cytotoxicity toward normal cells, thereby severely limiting its direct in vivo application. In contrast, PDA coating markedly improved these limitations, as the hemolytic risk of MnO_2_ was substantially reduced and cytotoxic effects on vascular and other normal cells were significantly alleviated, resulting in a pronounced enhancement of overall biocompatibility. In addition, the intrinsically low protein adsorption property of PDA reduces implant-associated immune rejection and inflammatory responses [59], further supporting its suitability for in vivo antioxidant applications. Beyond the intrinsic low toxicity of PDA toward biological systems, the safety and functional synergy of its degradation products are equally critical, as they further ensure the long-term in vivo antioxidant performance of PDA. Jin et al. [60] co-cultured macrophages with PDA degradation products, which primarily consisted of dopamine, quinone species, and PDA fragments, and observed no apparent cytotoxicity regardless of inflammatory stimulation, with consistently high cell viability. These findings indicate that PDA degradation products do not induce additional cellular damage, thereby reinforcing the biocompatibility profile of PDA. More importantly, these degradation products exert synergistic biological effects, including scavenging excess ROS generated under inflammatory conditions and alleviating oxidative stress, as well as attenuating inflammatory responses by modulating inflammation-related signaling pathways and reducing the release of pro-inflammatory cytokines. This provides an additional safety margin for the in vivo antioxidant therapeutic application of PDA by minimizing potential risks associated with material degradation. Overall, the high biocompatibility of PDA allows its direct use in biomedical applications without the necessity of additional delivery platforms, while simultaneously serving as an effective carrier for other bioactive agents. However, Li et al. [61] reported that high concentrations of PDA exert measurable effects on blood coagulation and complement activation, indicating that its application at elevated concentrations warrants further investigation.

## Regulation of Antioxidant Property

The intrinsic radical-scavenging capability of PDA arises from its chemical structure enriched with catechol/quinone moieties and amine groups. However, its antioxidant efficiency is often limited by the burial of active sites and insufficient environmental stability [62]. Through rational design strategies, PDA can exhibit enhanced performance in the treatment of oxidative stress-mediated diseases. Accordingly, this section discusses strategies to enhance the antioxidant performance of PDA, focusing on structural and morphological optimization, nanoparticle size regulation, and compositional modulation.

### Structural Modulation for Enhanced Antioxidant Activity

**Fig. 1:**
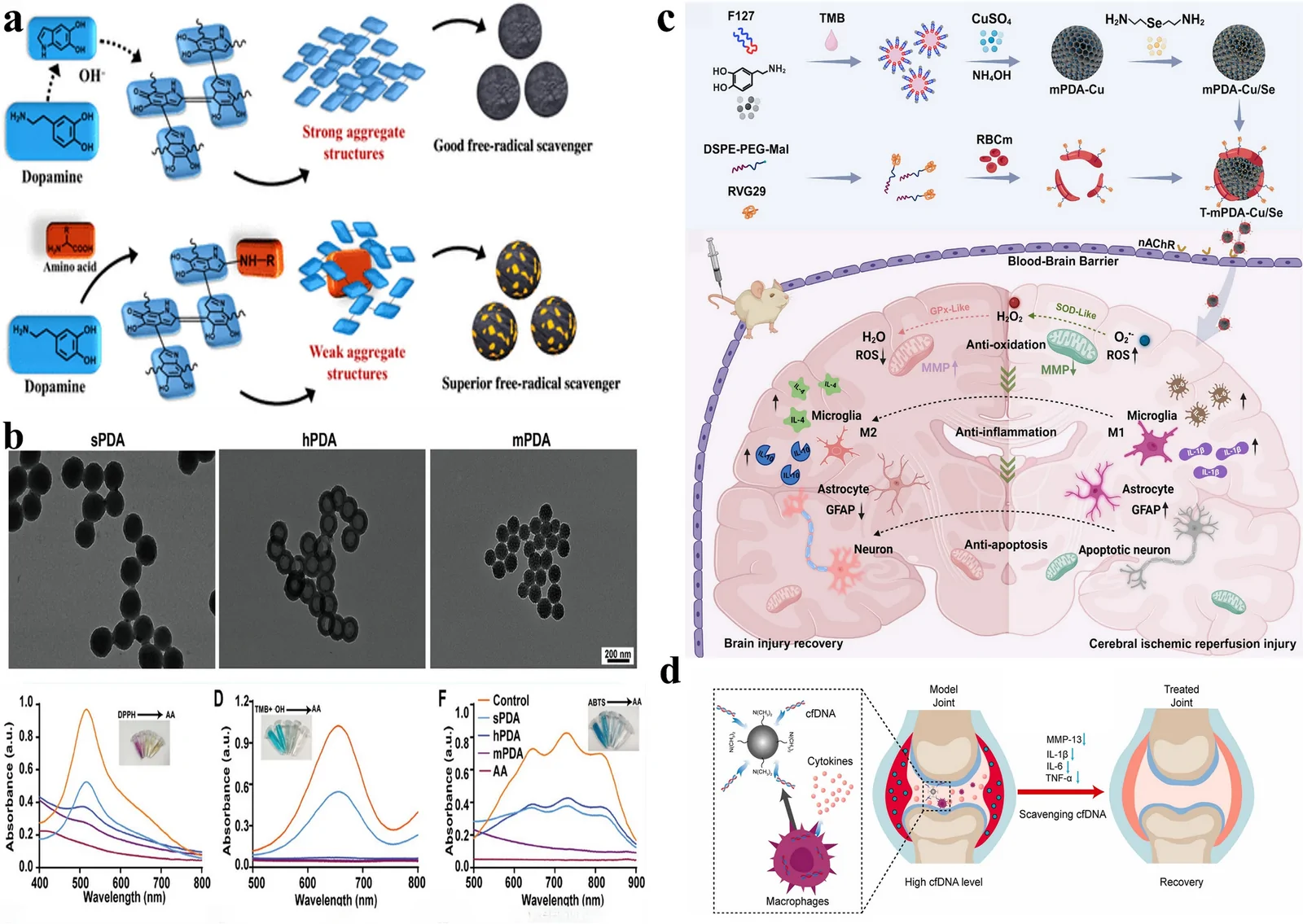
**a** Microstructural regulation strategies for tuning the free-radical scavenging capability of PDA. Reproduced with permission [64]. Copyright 2020, Chinese Chemical Society. **b** Transmission electron microscopy images of PDA nanoparticles with different morphology and comparison of their radical-scavenging activities. Reproduced with permission [70]. Copyright 2024, Wiley. **c** Cu/Se dual-element-doped MPDA exhibiting SOD- and GPx-mimicking activities. Reproduced with permission [75]. Copyright 2025, Wiley. **d** Strong binding of cationic PDA to cfDNA leading to reduced expression of pro-inflammatory cytokines. Reproduced with permission [83]. Copyright 2022, KeAi

During the oxidative polymerization of dopamine into PDA, multiple functional groups, including hydroxyl, amine, and quinone moieties, are generated, which endow PDA with intrinsic ROS-scavenging capability. However, excessive crosslinking can reduce the exposure of these active functional groups, thereby limiting their accessibility to ROS and ultimately diminishing the antioxidant activity of PDA [63]. Yang et al. [64] demonstrated that the copolymerization of dopamine with a basic amino acid (arginine) in aqueous solution induces arginine doping to disrupt the dense π–π stacking microstructure within PDA. The resulting arginine-doped melanin-like PDA nanoparticles exhibit a disordered, nonplanar internal microstructure (Fig. 1a), which facilitates the exposure of active sites and enhances radical scavenging and antioxidant performance. Nevertheless, this strategy is highly dependent on the compatibility between the dopant building units and melanin monomers, thus presenting certain limitations. To address this issue, Yang et al. [65] recently proposed a simple and effective approach to disrupt the dense π–π conjugated aggregation within melanin via condensation polymerization. Specifically, multiple condensation reactions were induced between the phenolic groups of 5,6-dihydroxyindole (DHI) and aldehydes (formaldehyde, acetaldehyde, or benzaldehyde) during the auto-oxidative radical polymerization process. The resulting melanin-like nanoparticles exhibited a loosely packed π-conjugated structure with reduced density, which further enhanced their antioxidant capability. Notably, the antioxidant performance of these melanin-like nanoparticles could be finely tuned by adjusting the chemical nature of the aldehyde linkers and the condensation ratio during polymerization. Beyond structural loosening, the antioxidant activity of PDA can be further enhanced indirectly by modulating its polymerization pathway to increase the overall density of accessible active functional groups within the structure. Recently, Rey et al. [66] compared PDA nanoparticles synthesized using three different alkaline agents: ammonium hydroxide, Tris, and Bicine. Fluorescence characterization revealed that, compared to PDA synthesized with ammonium hydroxide, the samples prepared with Tris and Bicine contained a higher relative content of amino and carbonyl groups, indicating distinct oxidation levels and structural evolution pathways. Correspondingly, these PDA samples exhibited superior scavenging efficiency in ROS quenching assays. These results demonstrate that the alkaline environment can modulate the antioxidant performance of PDA by governing its polymerization behavior, structural state, and electron-transfer kinetics.

Beyond the structural factors discussed above, modulation of the redox state of PDA represents another effective strategy to enhance its antioxidant activity. The synthesis of PDA is governed by intricate redox processes, including the oxidation of dopamine monomers to dopamine quinone, intramolecular cyclization, formation of indole derivatives, and crosslinking reactions among catechol units [67]. Consequently, the antioxidant capacity of PDA is, to a considerable extent, dependent on the degree of oxidation during polymerization. This redox state can be deliberately regulated through different preparation protocols. Liu et al. [26] incubated PDA with reducing agents such as ascorbic acid, NADPH, or glutathione to obtain a reduced form of PDA (PDA-red), followed by incubation in air-saturated water to generate an oxidized form of PDA (PDA-ox). Characterization results revealed that PDA-red contained a higher proportion of catechol moieties, whereas PDA-ox was enriched in quinone species derived from catechol oxidation. Subsequent experiments demonstrated that PDA-red exhibited significantly improved ROS-scavenging capacity along with pronounced anti-inflammatory effects. This study provides a new and effective perspective for improving the antioxidant performance of PDA through redox-state engineering.

### Size and Morphology Engineering

It is well established that particle size plays a critical role in determining the physicochemical properties of nanomedicines [68]. Similarly, the particle size of PDA has a pronounced impact on its antioxidant activity, and rational size regulation enables optimization of its antioxidant performance for diverse oxidative stress-mediated diseases. Alessio et al. [69] compared the radical-scavenging capacity of PDA nanoparticles with different sizes using hydroxyl radical-scavenging assays and demonstrated that reducing particle diameter markedly enhanced antioxidant efficiency, with an increase of up to 95.1%. This enhancement can be attributed to the increased specific surface area and the greater exposure of active functional groups on smaller nanoparticles. In addition, accumulating evidence suggests that smaller-sized PDA nanoparticles exhibit superior nanozyme-like antioxidant activity. For example, Han et al. [28] synthesized ultrasmall PDA nanoparticles that exhibited remarkable SOD- and CAT-mimicking activities, enabling efficient scavenging of reactive oxygen and nitrogen species and attenuation of cellular senescence.

Meanwhile, the nanostructural architecture of PDA nanoparticles also plays a decisive role in their antioxidant activity. Zheng et al. [70] synthesized three representative PDA nanostructures, including solid PDA, hollow PDA (HPDA), and mesoporous PDA (MPDA), and comparatively evaluated their intracellular ROS-scavenging capabilities. The results revealed that MPDA exhibited significantly higher ROS removal efficiency than the other PDA nanostructures (Fig. 1b). These findings indicate that a higher specific surface area confers enhanced free-radical scavenging activity to PDA. Previous studies have further demonstrated that employing appropriate oxidants during PDA synthesis can effectively increase surface roughness, thereby enlarging the accessible surface area [71]. In addition, recent studies have suggested that the two-dimensional ultrathin architecture endows PDA nanosheets with a high surface area and abundant phenolic groups, implying superior radical-scavenging and anti-inflammatory activities compared with conventional spherical PDA nanoparticles [72]. However, this hypothesis still requires further experimental validation.

### Compositional Engineering for Synergistic Antioxidant Effects

Modulating the compositional components of PDA-based nanoplatforms represents an effective strategy to regulate and enhance the antioxidant activity of PDA. First, owing to its excellent metal-ion chelation capability, PDA can be doped with various metal species to improve its overall antioxidant performance. For example, Song et al. [73] developed a functional hydrogel composed of Ag-integrated PDA nanoparticles via the self-assembly of dopamine and silver ions. The antioxidant activity of this system exhibited a concentration-dependent enhancement with increasing Ag incorporation. Moreover, when the incorporated metals possess intrinsic antioxidant nanozyme activity, they can synergize with PDA to form composite nanozymes, thereby further enhancing free-radical scavenging efficiency [74]. For instance, Wu et al. [75] achieved stable Cu loading through chelation between Cu ions and the catechol groups of PDA, followed by the introduction of Se via Schiff-base reactions and Michael addition, ultimately constructing a bimetallic cascade nanozyme (MPDA-Cu/Se). From a catalytic perspective, MPDA-Cu/Se mimics the cascade enzymatic activities of SOD and GPx, enabling robust and sustained ROS elimination (Fig. 1c).

Second, since the antioxidant capability of PDA can be partially attributed to its antioxidant enzyme-mimicking activity [76], enhancing this nanozyme behavior through the incorporation of additional components represents an effective approach to improve its overall antioxidant performance and radical-scavenging efficiency. It is well established that selenocysteine (SeCys) is essential for the antioxidant function of GPx. Accordingly, Wang et al. [77] co-polymerized PDA in the presence of SeCys to construct a GPx-active nanocomposite (PDA-SeCys). The results demonstrated that PDA-SeCys retained the intrinsic CAT- and SOD-like activities of PDA, while simultaneously exhibiting enhanced radical-scavenging efficiency and additional GPx-mimicking activity. In addition, because the catechol moieties of PDA are susceptible to oxidation [41], which compromises its antioxidant activity, incorporating protective components that stabilize catechol structures constitutes an effective strategy to preserve and enhance PDA antioxidant performance. For instance, Zhang et al. [78] prepared a hydrogel through free-radical polymerization of acrylated dopamine, acrylamide, and phenylboronic acid-modified dextran. In this system, dynamic boronate ester linkages between PDA catechol groups and phenylboronic acid efficiently prevented catechol auto-oxidation, thereby preserving the antioxidant functionality of PDA.

### Surface Charge Regulation for Enhanced ROS Affinity

Regulating the surface charge of PDA by introducing cationic moieties through surface modification represents an effective approach to enhance its antioxidant activity [79]. The PDA structure contains both protonatable amine groups and deprotonatable phenolic hydroxyl groups, rendering its surface potential highly sensitive to environmental pH variations. Under acidic conditions, PDA surfaces exhibit a positive charge, whereas under alkaline conditions they become negatively charged [80]. Positively charged PDA surfaces can effectively attract and enrich negatively charged ROS, such as O_2_·^−^, via electrostatic interactions, thereby facilitating their interaction with metal active sites or quinone moieties within PDA and significantly enhancing its SOD-like enzyme-mimicking activity [81]. Therefore, retaining or introducing positively charged functionalities, such as protonated amines or cationic polymer coatings, on PDA surfaces enhances their affinity toward anionic ROS, ultimately improving overall antioxidant performance.

Second, cationic PDA can indirectly contribute to antioxidant effects, for example, by electrostatically binding circulating free DNA (cfDNA) and thereby blocking its pathological activity. cfDNA consists of nuclear and mitochondrial DNA fragments predominantly released from necrotic cells and has been demonstrated to play a critical role in inflammatory diseases [82]. In inflammatory microenvironments, excessive cfDNA activates the TLR9-NF-κB signaling pathway, thereby inducing ROS generation. For instance, Chen et al. [83] modulated the positive surface charge density of PDA via surface modification with dimethylamino groups. The resulting cationic PDA nanoparticles exhibited strong binding affinity toward cfDNA and effectively suppressed cfDNA-induced inflammation, thereby improving therapeutic outcomes in rheumatoid arthritis (RA) (Fig. 1d). These findings indicate that chemical functionalization-induced surface cationization of PDA constitutes an effective strategy to enhance its overall antioxidant performance.

## Classification of PDA-Based Nanoplatforms

Through rational structural design and surface functionalization, PDA-based nanoplatforms have evolved into a diverse array of systems. In the following sections, these platforms are categorized into five major classes: solid, hollow, mesoporous, co-assembled, and coated systems, to elucidate their core characteristics while integrating the functional advantages conferred by surface modification. These structurally diverse and functionally synergistic PDA-based nanoplatforms, featuring tunable antioxidant activity, precise lesion targeting, and microenvironment responsiveness, offer innovative solutions that overcome the limitations of conventional therapeutic strategies for oxidative damage-related diseases (Table 1).

**Table 1:** Structure-dependent properties of PDA-based nanoplatforms

Morphology	Structural feature	Key antioxidant advantages	Therapeutic functionality
Solid PDA	Dense core, high stability	Surface HAT/ET; intrinsic SOD/CAT-mimicking activity	Prolonged, stable ROS scavenging for chronic inflammation where sustained antioxidant activity is prioritized over drug loading
MPDA	High surface area, ordered pore channels	Maximized active site exposure for enhanced HAT/ET; high SOD/CAT-mimicking activity; high drug loading capacity	Combinatorial therapy platform leveraging high payload capacity and sustained release for localized treatments requiring both drug delivery and intrinsic ROS scavenging
HPDA	Large inner cavity, tunable shell thickness	Surface HAT/ET; intrinsic SOD/CAT-mimicking activity; ultra-high drug loading capacity	High-capacity reservoir for severe inflammation requiring massive drug delivery, with the shell providing antioxidant protection
Doped PDA	Metal/non-metal incorporation into PDA matrix	Amplified/synergistic enzyme-mimicking activity	Enhanced catalytic ROS clearance for microenvironments with severe oxidative stress, often combined with imaging or other therapeutic modalities
PDA-coated nanoplatforms	Core–shell structure	PDA shell provides direct ROS scavenging + core contributes enzyme-mimicking activity	Versatile protective interface that improves biocompatibility and stimuli-responsiveness while preserving the core's primary function

### Basic PDA Nanoparticles

As the most fundamental form of PDA-based nanoplatforms, basic PDA nanoparticles can be readily fabricated into diverse structural morphologies solely by regulating the oxidative self-polymerization of dopamine. These morphologies mainly include three representative architectures: solid PDA nanoparticles, MPDA nanoparticles, and HPDA nanoparticles. Pronounced differences among these structures exist in terms of specific surface area, pore architecture, exposure of active functional groups, and microenvironment-responsive behaviors, thereby imparting distinct structure-dependent characteristics in antioxidant activity, drug-loading capacity, photothermal responsiveness, and in vivo delivery performance. In this subsection, the synthesis strategies and structural features of these three types of PDA nanoparticles are systematically summarized, with a particular emphasis on their advantages and potential applications in oxidative stress regulation, providing a structural foundation for the subsequent construction of doped and coated PDA-based nanoplatforms.

#### Solid PDA Nanoparticles

As the most fundamental nanostructure, solid PDA nanoparticles can be directly synthesized through the polymerization of dopamine. Currently, the most widely employed approach is the solution oxidation method. Under alkaline conditions, dopamine is oxidized to dopamine quinone in the presence of dissolved oxygen, followed by molecular rearrangement and intramolecular cyclization through a 1,4-Michael addition to form DHI. DHI and its quinone derivatives subsequently generate a variety of oligomeric species with different degrees of polymerization [84]. These oligomers can further self-assemble into PDA nanoparticles through a combination of covalent and noncovalent interactions [67]. As discussed above, the particle size of PDA nanoparticles has a pronounced influence on their antioxidant activity, making precise control over particle size of critical importance. At present, the most common strategy involves regulating the thermodynamic and kinetic parameters of dopamine polymerization to systematically modulate the ultimate particle size. By carefully adjusting factors such as pH, temperature, initial dopamine concentration, and reaction time, researchers can effectively suppress continuous nanoparticle growth and achieve predictable size control [69, 85, 86]. Distinct from parameter optimization, Huang et al*.* [87] proposed an alternative strategy for fine-tuning the size of PDA nanoparticles by introducing specific chemical derivatives to reversibly retard dopamine polymerization and precisely regulate the final particle size. Notably, it has also been reported that [88] prolonged ultrasonication can enhance PDA nucleation, leading to the formation of smaller-sized PDA nanoparticles, which provides additional possibilities for the controllable synthesis of PDA nanoparticles with tunable sizes.

**Fig. 2:**
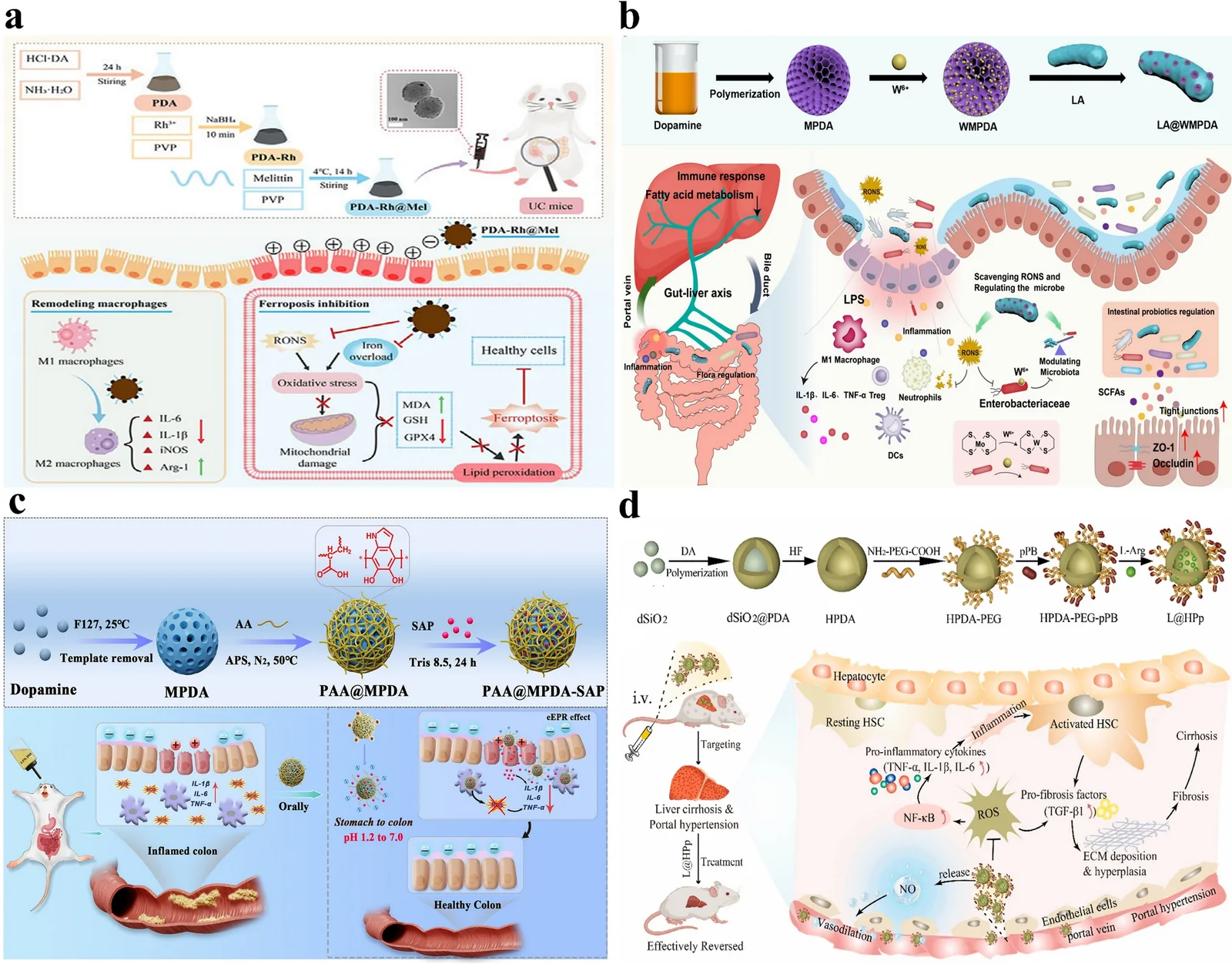
**a** Schematic illustration of the construction of PDA-Rh@Mel and its therapeutic mechanism in UC. Reproduced with permission [90]. Copyright 2025, Wiley. **b** Preparation of LA@WMPDA and its application in the treatment of IBD through modulation of the gut-liver axis. Reproduced with permission [100]. Copyright 2025, American Chemical Society. **c** PAA@MPDA-SAP enriched in colonic inflammatory sites to scavenge excessive ROS and release loaded SAP for synergistic IBD therapy. Reproduced with permission [106]. Copyright 2023, Elsevier. **d** Construction of L@HPp and its therapeutic effects in liver cirrhosis. Reproduced with permission [113]. Copyright 2023, Elsevier

Although solid PDA nanoparticles exhibit considerable potential in alleviating oxidative stress owing to their excellent antioxidant activity, favorable biocompatibility, and straightforward preparation processes [40, 89], their single therapeutic modality represents a major bottleneck for further clinical translation. Consequently, surface drug adsorption and functional modification are commonly employed to enhance their therapeutic efficacy. For example, in the treatment of ulcerative colitis (UC), Li et al. [90] developed a multifunctional nanozyme, PDA-Rh@Mel, loaded with melittin. The amine groups on the PDA surface coordinated with Rh^3^⁺ ions, which were subsequently reduced in situ by NaBH₄ to generate Rh aggregates (PDA-Rh), thereby introducing CAT activity and enabling SOD-CAT cascade enzymatic catalysis for efficient ROS scavenging. Meanwhile, melittin was immobilized via electrostatic adsorption between the negatively charged PDA surface and the positively charged Mel, which preserved the anti-inflammatory and immunomodulatory functions of Mel while effectively mitigating the hemolytic toxicity associated with free Mel (Fig. 2a). In addition, surface modification endows PDA nanoparticles with enhanced targeting capability, allowing the construction of more precise functional nanoplatforms. Macrophages are key participants in inflammatory processes and possess intrinsic chemotactic properties toward inflammatory sites, primarily attributed to chemokine receptors embedded in the macrophage membrane (MM), such as CCR2 and CXCR1 [91]. These receptors function as molecular gateways that facilitate macrophage adhesion to inflamed tissues, thereby promoting host defense responses. Bao et al. [92] conjugated PDA nanoparticles with the antimicrobial peptide mCRAMP and subsequently coated them with MMs to obtain MMs/PDA/mCRAMP nanoparticles. Owing to the inflammation-targeting properties of activated MMs, the activated MM was further employed as the outermost coating layer, enabling active targeting of inflamed tissues while synergistically exerting ROS-scavenging and immunomodulatory effects. Moreover, polyethylene glycol (PEG) modification can effectively improve the aqueous dispersibility and in vivo stability of solid PDA nanoparticles, reduce serum protein adsorption and immune clearance, and, in the context of osteoarthritis (OA) therapy, decrease particle aggregation while enhancing ROS-scavenging capability [93].

#### MPDA Nanoparticles

As discussed above, MPDA exhibits the most prominent ROS-scavenging capability among different PDA nanostructures owing to its exceptionally high specific surface area. This structural advantage simultaneously endows MPDA with superior drug-loading capacity [94, 95], thereby substantially broadening its applications in oxidative stress-mediated diseases. The synthesis of MPDA nanoparticles is adapted from classical mesoporous material fabrication strategies in which soft templating agents are employed alongside pore-expanding molecules to guide PDA polymerization at defined interfaces. Upon the subsequent extraction of the structural templates, the resulting MPDA nanoparticles exhibit well-organized mesoporous architectures and significantly expanded specific surface areas [96, 97]. The clinical efficacy of many anti-inflammatory and antioxidant therapeutics is constrained by suboptimal water solubility, limited formulation stability, and rapid clearance following systemic administration, which severely limit their clinical application. MPDA-based delivery systems provide an effective solution to these challenges. For instance, oral probiotic administration has demonstrated notable therapeutic efficacy, reduced toxicity, fewer adverse effects, and improved patient compliance in the treatment of inflammatory bowel disease (IBD) [98]. However, many probiotics lose viability before reaching the intestine due to the highly acidic gastric environment, digestive enzymes, and elevated ROS levels in pathological conditions [99]. To address this issue, Deng et al. [100] utilized MPDA encapsulation to protect probiotics during gastrointestinal transit. In their designed therapeutic system, LA@WMPDA (tungsten ion-loaded MPDA encapsulating Lactobacillus acidophilus), the MPDA shell effectively shielded the probiotics from gastric acidity and intestinal digestive enzymes, thereby significantly enhancing bacterial survival and overcoming a key limitation of oral probiotic therapy. Meanwhile, the abundant catechol groups on the MPDA surface enabled strong adhesion to the probiotic surface, forming a stable LA@WMPDA biohybrid structure without compromising bacterial viability (Fig. 2b). Furthermore, NIR-responsive strategies can exert synergistic therapeutic effects in oxidative stress-mediated diseases through photothermal conversion and controlled drug release. Owing to its large surface area and mesoporous architecture, MPDA exhibits higher photothermal conversion efficiency than solid PDA nanoparticles, while displaying a lower cumulative drug release profile, thereby enabling more pronounced sustained-release behavior [101].

To further enhance targeting precision and therapeutic synergy, surface-modified MPDA nanoplatforms have demonstrated more comprehensive functional advantages. In the treatment of brain-related diseases, the presence of the blood–brain barrier (BBB) severely restricts the delivery of many drugs and biological agents to the central nervous system, posing substantial challenges for both diagnosis and therapy. Peptides [102], owing to their high specificity, low immunogenicity, and favorable tissue penetration capability, are frequently employed to improve the BBB permeability of nanoparticles. For instance, RAP-12 displays strong binding affinity for low-density lipoprotein receptor-related protein-1, a receptor highly enriched on the surface of brain microvascular endothelial cells constituting the BBB. By specifically recognizing and binding to low-density lipoprotein receptor-related protein-1, RAP-12 can trigger receptor-mediated endocytosis in BBB endothelial cells. Accordingly, Wu et al. [103] conjugated RAP-12 onto the surface of MPDA nanoparticles using a thiol-terminated PEG polymer as a linker to construct a brain-targeted nanoplatform. Experimental results showed that, compared with the non-targeted system, RAP-12-modified nanoparticles achieved a 451% increase in fluorescence intensity within the brain. This markedly enhanced the therapeutic efficacy of PDA-based nanoplatforms in brain oxidative stress-related diseases. In addition, polyacrylic acid (PAA), a representative pH-responsive polymer, has been widely utilized to modify and functionalize various nanocarriers for therapeutic applications [104, 105]. PAA can be grafted onto the surface of PDA nanoparticles via coupling between its carboxyl groups and the amine groups of PDA, and the introduction of abundant carboxyl moieties significantly enhances the pH responsiveness of PDA nanoplatforms. When the environmental pH is lower than the pKa of PAA, the polymer chains remain in a collapsed and neutral state, tightly coating the PDA surface; conversely, when the pH exceeds the pKa of PAA, the carboxyl groups become deprotonated, rendering the polymer chains negatively charged and highly swollen. For instance, Guan et al. [106] fabricated a PAA-coated MPDA-SAP nanocarrier (PAA@MPDA-SAP), in which the PAA layer effectively prevented degradation and premature drug release in the acidic gastric environment following oral administration, while enabling rapid drug release at inflamed colonic sites. Moreover, owing to the abundance of positively charged proteins in inflamed colonic tissues, the negatively charged PAA@MPDA-SAP nanoparticles could preferentially accumulate in inflamed colon regions, thereby further improving therapeutic efficacy (Fig. 2c).

#### HPDA Nanoparticles

In the preparation of HPDA nanostructures, sacrificial templates such as silica microspheres are commonly employed to first form a core–shell architecture, followed by selective template etching [107]. Because traditional harsh etchants such as hydrofluoric acid may compromise the biological activity of encapsulated payloads alternative mild approaches including water-dissolution strategies [108] or the use of water-removable sodium citrate cores [109] have been developed to achieve efficient encapsulation without residual contamination. Structurally, unlike the multi-pore honeycomb architecture characteristic of MPDA, HPDA features a single hollow cavity enclosed by a PDA shell. This hollow configuration endows HPDA with superior payload capacity and sustained-release behavior [110]. Furthermore, the shell thickness of HPDA can be precisely tuned by adjusting the dopamine monomer concentration during synthesis [111]. A thicker shell prolongs the drug diffusion path, resulting in slower release, while a thinner shell enables faster release. This thickness-dependent release kinetics provides an additional strategy for tailoring therapeutic regimens to specific disease microenvironments. Moreover, the excellent pH responsiveness and photothermal conversion capability of HPDA enable precise, on-demand drug release and targeted delivery. For instance, in the treatment of diffuse alveolar hemorrhage, Zhang et al. [112] encapsulated the immunosuppressant cyclophosphamide (CTX) within HPDA to construct a CTX@HPDA nanoplatform. In this system, HPDA facilitated the targeted transport of CTX to inflamed tissues under NIR irradiation and pH stimulation, followed by photothermally triggered drug release.

To further enhance targeting precision and therapeutic efficacy, surface-modified HPDA nanoparticles exhibit enhanced translational potential by integrating active targeting with sustained-release capability, thereby enabling precise and long-lasting antioxidant therapy. For example, Wang et al. [113] encapsulated L-arginine (L-Arg) within HPDA and subsequently functionalized the surface with pPB peptides for the synergistic treatment of liver fibrosis (LF). Benefiting from its unique hollow architecture, HPDA achieved high L-Arg payloads, with a loading capacity of up to 38.9%. Meanwhile, surface-decorated pPB peptides enabled active targeting toward hematopoietic stem cells, resulting in prolonged hepatic retention of the nanoplatform for up to 14 days and a pronounced reversal of LF. Furthermore, HPDA modulated the hepatic redox microenvironment, thereby facilitating L-Arg-derived NO production. The elevated NO levels synergistically promoted portal vein vasodilation, alleviated portal hypertension, and ultimately contributed to effective anti-fibrotic therapy (Fig. 2d).

### Doped PDA Nanoparticles

**Fig. 3:**
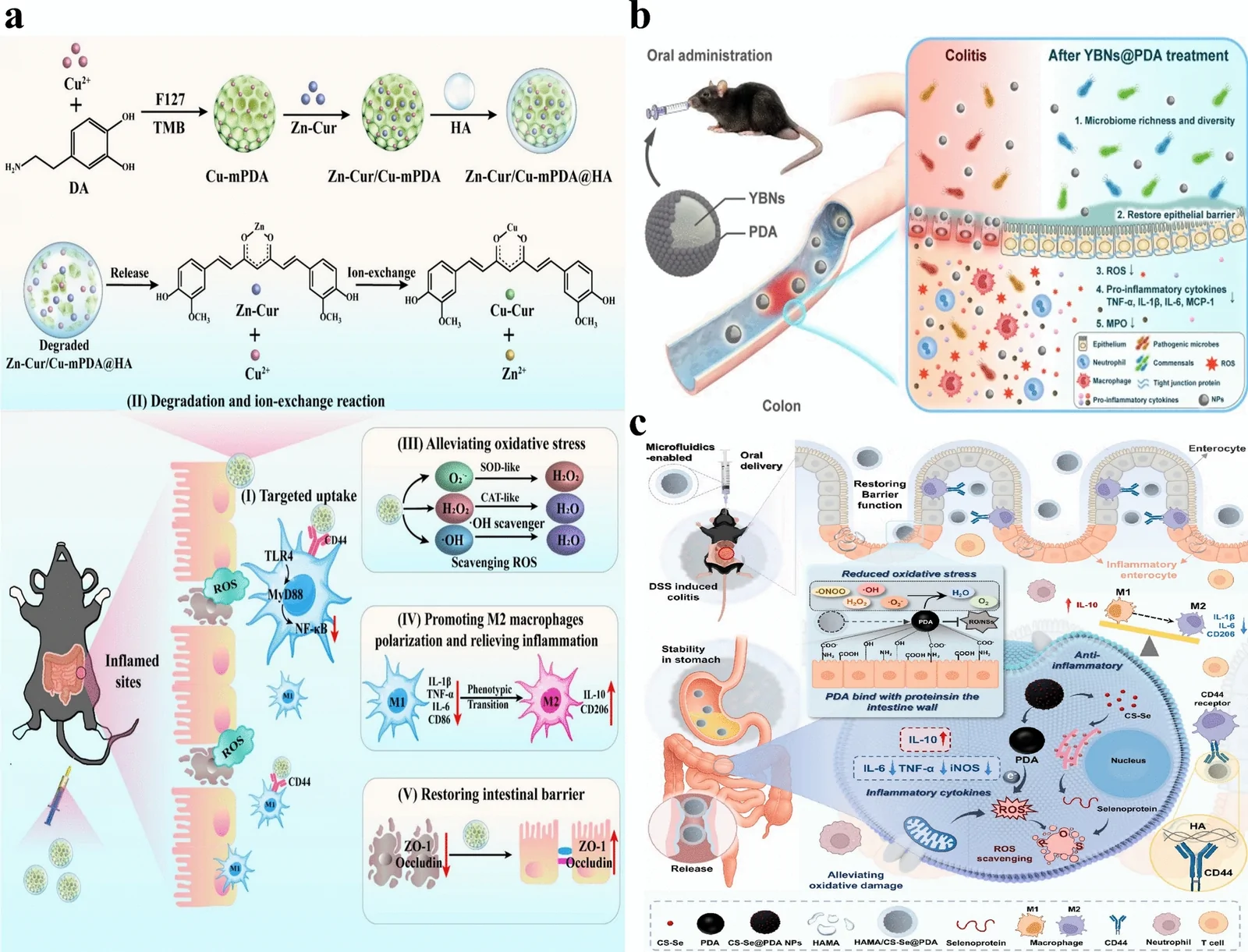
**a** Schematic illustration of the synthesis of Zn-Cur/Cu-MPDA and its therapeutic mechanism in alleviating colitis. Reproduced with permission [114]. Copyright 2025, Elsevier. **b** Design of YBNs@PDA and its application for IBD treatment. Reproduced with permission [125]. Copyright 2024, Springer Nature. **c** Oral administration of HAMA/CS-Se@PDA hydrogel microspheres and their therapeutic mechanism in colitis. Reproduced with permission [127]. Copyright 2025, Elsevier

Doped PDA nanoparticles not only exhibit enhanced antioxidant performance at the molecular level but also provide a highly engineerable nanoplatform for constructing multifunctional therapeutic delivery systems. Benefiting from abundant surface chemical moieties, excellent nucleation stability, and favorable biocompatibility, PDA enables the in situ incorporation of metal or non-metal species during polymerization, yielding structurally stable and highly integrated nanostructures. Compared with conventional nanomaterials, doped PDA nanoplatforms demonstrate superior performance in electron-transfer efficiency, microenvironment responsiveness, and theranostic integration. Among various strategies, metal doping represents one of the most widely adopted and effective approaches to enhance PDA functionality. Owing to abundant catechol and amine moieties [74], PDA can stably chelate a broad range of metal ions. For example, Lu et al. [114] prepared copper-doped mesoporous PDA (Cu-MPDA) by co-polymerizing dopamine with Cu^2^⁺ for the treatment of IBD. Compared with pristine MPDA, Cu-MPDA exhibited a pronounced enhancement in antioxidant capacity, which was attributed to Cu-induced amplification of SOD-like and CAT-like activities (Fig. 3a). Beyond antioxidant enhancement, selected metal dopants can additionally endow PDA nanoplatforms with imaging functionality, which is of particular significance for inflammatory diseases such as arthritis and atherosclerosis. For instance, Mn^2^⁺ [115] and Gd^3^⁺ [116], as T1-weighted magnetic resonance imaging (MRI) contrast agents, enable noninvasive MRI tracking when incorporated into PDA nanoparticles. This imaging capability allows real-time visualization of inflammation progression, thereby providing a valuable basis for disease diagnosis and therapeutic monitoring.

Beyond metal doping, non-metal doping can also substantially improve the therapeutic efficacy of PDA nanoparticles in oxidative stress-mediated diseases. PDA is well recognized as an efficient photothermal agent, and its NIR responsiveness has been widely explored for the treatment of inflammatory and neurodegenerative disorders. However, when PDA is used alone as a photothermal agent, relatively high laser power is often required to achieve therapeutic temperatures, which may result in collateral thermal damage to surrounding normal tissues [117]. A recent study [118] has demonstrated that nitrogen-doped carbon quantum dots (CQDs) possess favorable absorption and emission spectra, enabling more efficient light harvesting and enhanced photothermal conversion efficiency. By integrating CQDs, PDA can capture additional photonic energy and achieve effective photothermal therapy under reduced laser power density. Accordingly, in an Alzheimer’s disease (AD) model, Liu et al. [119] constructed a PDA-CQD composite nanoplatform. The incorporation of CQDs effectively mitigated thermal damage to normal brain tissue and neurons under high-power laser irradiation, allowing PDA to reach the same therapeutic temperature at significantly lower laser power. Furthermore, to enhance in vivo targeting and prolong systemic circulation, the PDA-CQD nanocomposites were camouflaged with red blood cell (RBC) membranes (PDA-CQD/RBC). This biomimetic RBC coating improved the targeted delivery of the nanoplatform to diseased brain regions, thereby synergistically enhancing photothermal efficiency and therapeutic safety.

Building upon these advances, an increasing number of studies have adopted dual-doping strategies to construct composite PDA nanoplatforms with multimodal therapeutic functions. For instance, Liu et al. [115] developed Mn^2^⁺/ Arg dual-doped PDA nanoparticles, in which the antioxidant activity of PDA was enhanced through two complementary mechanisms: structural loosening of the PDA framework and metal-catalyzed redox reactions. Meanwhile, the incorporation of Mn^2^⁺ endowed the nanoplatform with imaging capability, enabling visualization of diseased lesions and facilitating diagnostic guidance. Collectively, through synergistic doping strategies involving metal, non-metal, or their combinations, PDA nanoparticles can be significantly reinforced in both structural and functional dimensions. Such rationally engineered PDA-based nanoplatforms form highly efficient antioxidant systems and provide a robust materials foundation for the precise treatment of oxidative stress-mediated diseases.

### PDA-Coated Nanoplatforms

Benefiting from its outstanding bioinspired adhesive properties, PDA can form uniform and conformal coatings on the surface of diverse nanomaterials, enabling the construction of core–shell drug delivery systems. Such architectures preserve the intrinsic therapeutic functions of the core nanoparticles while integrating the physicochemical advantages of PDA, thereby enhancing therapeutic efficacy against oxidative stress-mediated diseases. Consequently, PDA-coated nanoplatforms have emerged as one of the most actively explored strategies in PDA-based drug delivery systems. First, although many natural or synthetic antioxidant agents, such as curcumin [120], resveratrol [121], and melatonin [55], exhibit well-established ROS-scavenging and anti-inflammatory activities, their clinical translation is often hindered by poor bioavailability and limited targeting efficiency. PDA coatings can encapsulate drug-loaded nanoparticles through physical adsorption or chemical conjugation, acting as a protective shell while endowing additional functionalities [122]. Under oxidative stress conditions, the catechol groups in PDA are oxidized into quinone structures, leading to weakened intermolecular interactions and subsequent disassembly of the PDA shell. This ROS-responsive behavior enables site-specific drug release at pathological regions. For example, He et al. [123] encapsulated the antioxidant edaravone (Eda) into diselenide-bridged mesoporous silica nanoparticles and subsequently coated the nanocarriers with PDA to obtain PDA-DSeMSN@Eda. In this system, the PDA shell functioned as a “gatekeeper” to prevent premature drug leakage during circulation. Upon exposure to a ROS-rich microenvironment, the PDA shell underwent oxidative degradation, triggering nanoparticle disassembly and controlled release of Eda. In addition, PDA coatings are particularly advantageous for gastrointestinal disease treatment owing to their strong mucoadhesive properties, which prolong intestinal retention time. Notably, PDA exhibits zwitterionic characteristics and can engage electrostatically with cationic choline moieties on epithelial cell membranes [124], facilitating mucus penetration and enhancing cellular uptake. For instance, Yang et al. [125] reported the development of a PDA-coated prebiotic yeast β-glucan nanocomposite (YBNs@PDA) for IBD therapy. The PDA shell enabled firm adhesion of YBNs to inflamed colonic mucosa, significantly extending intestinal residence time and improving drug bioavailability. Meanwhile, the PDA coating synergistically suppressed inflammatory responses by reducing pro-inflammatory cytokine levels, thereby further enhancing the therapeutic efficacy (Fig. 3b).

Nanozymes (e.g., CeO_2_ [126], MnO_2_ [58], and Se [127] nanoparticles), which exhibit prominent SOD-, CAT-, and peroxidase (POD)-mimicking activities, have emerged as promising substitutes for natural antioxidant enzymes owing to their high stability and low cost. However, their poor biocompatibility remains a major concern, significantly limiting biomedical applications. PDA coatings can effectively address this issue through surface modification and interfacial chemical interactions while simultaneously enhancing the antioxidant performance of nanozymes. Specifically, PDA coatings act as a physical barrier that minimizes direct contact between inorganic nanozymes and the biological environment. Moreover, owing to its melanin-like structure, PDA endows the resulting composite systems with improved biocompatibility and reduced cytotoxicity. For instance [128], CeO_2_ nanozymes tend to accumulate in the lungs and induce inflammatory responses during acute lung injury (ALI) treatment. PDA coating not only markedly reduces their cytotoxicity toward pulmonary epithelial cells but also decreases serum protein adsorption through surface catechol groups, thereby lowering monocyte-mediated phagocytosis and prolonging systemic circulation time. In addition, metal–organic frameworks (MOFs), characterized by high specific surface area, tunable pore structures, and intrinsic enzyme-mimicking activity, have also been explored as inorganic nanozymes for antioxidant therapy [129]. Nevertheless, MOF-based nanozymes often suffer from in vivo aggregation and short residence time. To overcome these limitations, Ge et al. [130] developed a PDA-coated zirconium-based MOF (Zr-MOF) Mn/NAC nanozyme for the treatment of cisplatin-induced hearing loss. The PDA shell significantly reduced the cytotoxicity of Zr-MOF-Mn and improved its biocompatibility. Meanwhile, the strong adhesive property of PDA enabled prolonged retention of the nanozyme in the middle ear, thereby enhancing targeted delivery to the inner ear. More importantly, PDA-mediated physical encapsulation of the Mn/NAC nanozyme ensured the structural integrity and in vivo stability of the microcapsule system.

Beyond the direct use of PDA as a coating layer, researchers have frequently introduced secondary chemical modification or surface functionalization onto PDA shells to construct composite nanoplatforms with multi-responsive and multimodal therapeutic capabilities. Li et al. [131] developed a multifunctional MnO_2_@PDA-based composite nanoplatform (MPMAF) by further decorating the PDA shell with folic acid (FA) and TNFα-aptamer, thereby enabling dual targeting of macrophages and inflammatory cytokines. In addition, for the treatment of UC, hyaluronic acid (HA), as a natural ligand of cluster of differentiation 44 (CD44), can selectively target inflamed colonic epithelial cells and activated immune cells [132], significantly enhancing local therapeutic efficacy. Based on this strategy, Fang et al. [127] embedded PDA-coated selenium nanoparticles into HAMA microspheres to fabricate HAMA/CS-Se@PDA hydrogel microspheres. In this system, the PDA coating contributed to restoring redox homeostasis by scavenging excess ROS and supporting epithelial barrier repair, while the HAMA shell provided pH-responsive protection and CD44-mediated targeting toward inflamed colonic tissues. The synergistic integration of these components markedly enhanced mucosal adhesion, inflammation-targeted delivery, and coordinated redox regulation, thereby establishing a precise therapeutic platform for UC (Fig. 3c). Collectively, PDA-based composite nanoplatforms constructed through secondary functionalization combine the biomimetic antioxidant properties of PDA with the synergistic effects of exogenous functional components, offering a flexible and highly efficient therapeutic strategy for addressing complex oxidative stress-mediated diseases.

## PDA-Based Nanoplatforms in Oxidative Stress Diseases

Although oxidative stress-related diseases encompass diverse tissues and disease modalities, they frequently share several core pathological hallmarks. These include the mutual amplification of ROS and inflammatory signals, the restriction of nanodelivery by biological barriers, the impairment of regenerative microenvironments, and the aberrant activation of programmed cell death. In recent years, nanomaterials have achieved remarkable progress in the fields of inflammation regulation [18], macrophage metabolic reprogramming [133], and ferroptosis [134], providing critical references for the design of PDA-based nanoplatforms. Therefore, this section systematically categorizes PDA-based nanoplatforms around these key pathological challenges. By abstracting generalizable engineering logic from specific application paradigms, we aim to provide a more instructive framework for the rational construction of materials tailored to diverse pathological scenarios (Table 2).

**Table 2:** Summary of strategy-driven PDA-based nanoplatforms for antioxidant therapy

Nanoplatforms	PDA morphology	Diseases model	Design strategy	Key mechanism	Refs
PPM	PDA Coating	Periodontitis	NIR-responsive release	ROS scavenging; NIR-triggered drug release; efficiently adsorbed Mino	[135]
Met@ZIF-8@PDA	PDA Coating	Periodontitis	Macrophage polarization regulation	ROS scavenging; M1 → M2 polarization; TNF-α/IL-6↓; delayed the release of Met and Zn2⁺	[136]
PDA/BBR@Gel@BMP9-PDLSC	Solid PDA	Periodontitis	Structural scaffold support	Sustained BBR release; enhanced tissue adhesion; microenvironment remodeling	[137]
MPM@Lipo	Solid PDA	RA	Pathway inhibition	ROS scavenging; photothermal ablation; NF-κB suppression	[138]
MPZH	HPDA	RA	Macrophage polarization regulation	Zn2⁺ coordination-enhanced SOD-like activity; NF-κB inhibition	[139]
COF-PDA	PDA Coating	OA	NIR-responsive release	NIR-controlled drug release; improved lubrication stability	[140]
SiO_2_@PP-Cur	PDA Coating	OA	Microenvironment remodeling	PMPC-induced friction reduction; ROS-responsive drug release; M1 → M2 polarization	[141]
LBL-CO@MPDA	MPDA	IBD	Gut microbiota modulation	CO prodrug delivery; ROS scavenging; restoration of gut immune homeostasis	[142]
CAT@ALG–PDA	PDA Coating	IBD	Mucoadhesive retention	ROS scavenging; catechol-mediated mucin adhesion; prolonged intestinal retention	[143]
TA-Met-PNP	MPDA	IBD	Gut microbiota modulation	ROS scavenging; Fe3⁺ coordination; inflammatory site targeting	[144]
MPO	MPDA	LF	Pathway inhibition	ROS scavenging; inhibition of TGF-β/SMAD signaling	[145]
L@HPp	HPDA	LF	Microenvironment remodeling	ROS scavenging; reduction of lipid peroxidation; hepatic stellate cells inhibition	[113]
K8@Fe-Rh/PDA	Solid PDA	AD	BBB penetration via peptide modification	Aβ-targeting peptide; ROS scavenging; inhibition of Aβ aggregation	[146]
PDA-CQD/RBC	Doped PDA	AD	Biomimetic camouflage	Disassembled Aβ fibrils; RBC membrane coating; NIR-assisted BBB penetration	[119]
SA-BP-MB/BBR	Solid PDA	AD	NIR-responsive release	NIR-triggered BBB opening and Aβ fibril disaggregation promoting; Cu2⁺ chelation	[147]
CPUD gel	PDA Coating	PD	Electroconductive microenvironment regulation	M1 → M2 polarization; ROS scavenging; TH + neuron protection	[148]
PDASeCys	Doped PDA	PD	Apoptosis attenuation	GPx-like activity; mitochondrial membrane stabilization	[77]
CPO/D@P/IGF-1	Solid PDA	Diabetic wounds	Promoting angiogenesis	DFO loading; HIF-1α stabilization; VEGF↑; M2 polarization	[149]
CBGT@PDA	Solid PDA	Diabetic wounds	Macrophage polarization regulation	ROS scavenging; inhibition of M1 polarization; angiogenesis promotion	[150]
RL-QN15-MPDA	MPDA	Burn wounds	Sustained release	Protected RL-QN15 from enzymatic degradation; prolonged local release	[151]
rGO/PDA/Ag/COT	PDA Coating	Burn wounds	Promoting angiogenesis	ROS scavenging; VEGF/TGF-β1 upregulation	[152]
T-mPDA-Cu/Se	MPDA	IRI	BBB penetration via peptide modification	RVG29-mediated BBB transport; cascade nanozyme ROS scavenging	[75]
PPR	Solid PDA	IRI	ROS-responsive release	ROS scavenging; mitochondrial permeability transition pore regulation; GPX4 upregulation	[153]
NDS	Solid PDA	SCI	Exosome-mediated transcytosis	Scavenged ROS; Neutrophil membrane vesicle-assisted CNS delivery	[154]
MPDA@8G	MPDA	SCI	Ferroptosis inhibition	Lipid peroxidation suppression; ferroptotic signaling inhibition	[155]
P@Co	Solid PDA	ALI	Macrophage polarization regulation	Nanozyme synergy; M1 → M2 polarization; HSP70 upregulation	[156]
CPPC	Solid PDA	ALI	Pathway inhibition	pH-responsive release; NF-κB suppression	[157]
GelMA-imid/SDF-1α/hAMSCs	Solid PDA	TBI	Providing structural scaffolds	hAMSC directional differentiation; Nissl body preservation; NeuN and BDNF upregulation	[158]
PDA-AMSN-D	PDA Coating	TBI	pH-responsive release	CAT-like activity; pH-triggered drug release; mitochondrial protection	[159]
MPDA@Mel-Tav	MPDA	DED	Mucoadhesive retention	Sustained dual-drug release; prolonged ocular surface retention	[160]
PDA@CNO	PDA Coating	IVDD	Structural scaffold support	Nanozyme synergy; enhanced tissue retention	[161]
PPSK	Solid PDA	AKI	KIM-1-targeted gene therapy	Klotho restoration; PPARα/FAO upregulation; lipid peroxidation↓; ROS scavenging	[162]
NPANs-MSCs	PDA Coating	Stroke	Regenerative microenvironment remodeling	ROS scavenging; ER-Ca2⁺-mitochondrial pathway regulation; VEGF and CD34 upregulation	[163]
RVG-SPIO-125I-PDA@EVs	PDA Coating	Stroke	RVG29-mediated neuronal targeting	PDA-enabled multifunctional engineering; ROS scavenging; targeted neuronal delivery; apoptosis↓	[164]

*PD* Parkinson’s disease, *IRI* ischemia–reperfusion injury, *TBI* traumatic brain injury, *DED* dry eye disease, *IVDD* intervertebral disc disease, *VEGF* vascular endothelial growth factor

### Breaking the Self-Amplifying ROS-Inflammation Feedback Loop

A central hallmark of many oxidative stress-related diseases is the self-amplifying loop between excessive ROS generation and chronic inflammation. Overproduced ROS trigger the activation of redox-sensitive transcription factors, such as NF-κB, leading to the secretion of pro-inflammatory cytokines, which subsequently exacerbates mitochondrial dysfunction and perpetuates ROS generation [165–167]. This vicious cycle drives the pathological progression of a broad spectrum of diseases, ranging from periodontitis and arthritis to IBD and LF. PDA-based nanoplatforms possess distinct advantages that enable the disruption of this cycle through multiple interconnected strategies.

#### Macrophage Polarization Regulation

In oxidative stress-related diseases, the imbalance of macrophage phenotypes represents a critical factor driving the persistent amplification of inflammation. Excessive ROS levels promote the polarization of macrophages toward the pro-inflammatory M1 phenotype and trigger the release of inflammatory factors such as TNF-α and IL-1β, which further exacerbates tissue damage. Consequently, regulating the polarization state of macrophages to inhibit M1-type inflammatory responses and promote the formation of the M2-type reparative phenotype has emerged as a significant design strategy for disrupting the ROS-inflammation amplification loop [133]. In this process, PDA functions not only as a ROS scavenger to alleviate oxidative stress but also as a regulator of the intracellular signaling environment to facilitate immune phenotype remodeling [168].

**Fig. 4:**
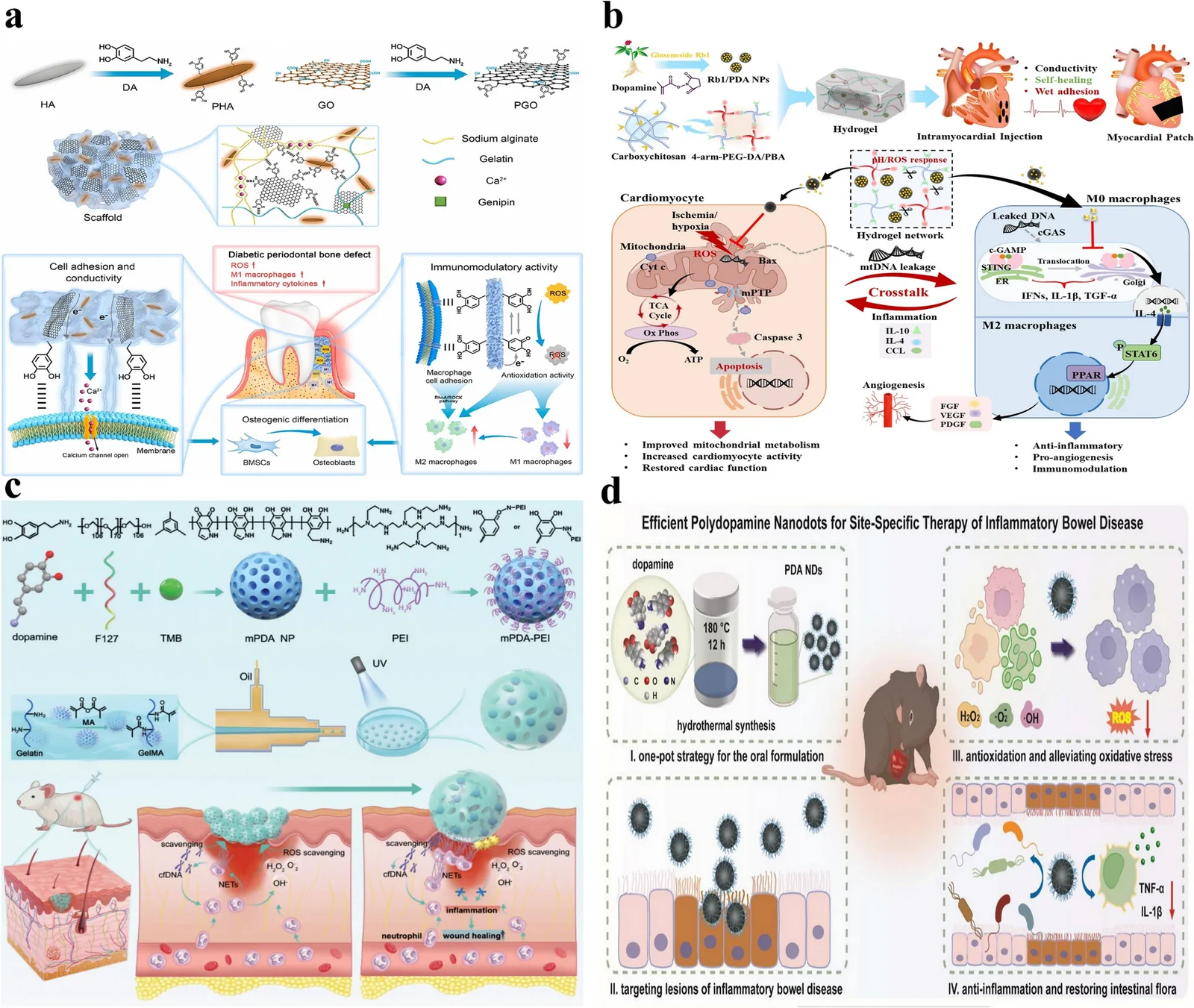
**a** Schematic illustration of PDA-mediated conductive scaffold promoting M2 macrophage polarization and osteogenesis-related cytokine secretion through immunometabolism and cytoskeletal remodeling. Reproduced with permission [170]. Copyright 2022, KeAi. **b** Rb1-loaded PDA hydrogel inhibits cGAS-STING pathway activation by scavenging ROS, alleviating inflammatory damage in myocardial infarction. Reproduced with permission [175]. Copyright 2024, Elsevier. **c** MPDA-PEI@GelMA hydrogel microspheres simultaneously scavenge cfDNA and ROS, reducing pro-inflammatory cytokine secretion in diabetic wounds. Reproduced with permission [179]. Copyright 2024, Wiley. **d** Ultrasmall PDA NDs restore gut microbiota homeostasis and alleviate inflammation in IBD. Reproduced with permission [182]. Copyright 2025, Wiley

To begin with, research has demonstrated that PDA itself possesses intrinsic immunomodulatory activity independent of loaded therapeutics. In the context of periodontitis treatment, Bai et al*.* [169] developed PDA-functionalized mesoporous silica nanoparticles and proved that the PDA shell alone could directly induce the transition of macrophages from M1 to M2, leading to a reduction in TNF-α and IL-6 levels and an increase in IL-10 secretion. This effect was independent of the co-loaded minocycline hydrochloride and emphasized the inherent biological activity of PDA. This suggests that PDA can actively remodel the inflammatory microenvironment by directly influencing macrophage polarization even in the absence of additional immunomodulatory agents. Beyond the aforementioned examples, studies have uncovered novel mechanisms by which PDA regulates macrophage polarization. Li et al*.* [170] developed a PDA-mediated graphene oxide and nanohydroxyapatite conductive scaffold for diabetic periodontal bone regeneration. This scaffold utilized the catechol groups of PDA to scavenge ROS and downregulate the HIF-1α-mediated glycolytic pathway, which inhibited M1 macrophage polarization. Simultaneously, the PDA on the scaffold surface promoted cell adhesion and the formation of pseudopodia, which activated the RhoA/ROCK signaling pathway to induce M2 macrophage polarization and the secretion of osteogenesis-related cytokines including BMP-2 and TGF-β1 (Fig. 4a). This research revealed a dual mechanism by which PDA affects macrophage phenotypes through the modulation of immunometabolism and cytoskeletal remodeling.

Building upon this intrinsic activity, researchers have further developed strategies to amplify the immunomodulatory effects of PDA by integrating it with bioactive substances. In the treatment of OA, Li et al*.* [171] constructed PDA@Exos microneedles to deliver bone marrow mesenchymal stem cell-derived exosomes into synovial tissues. Here, PDA functioned as a delivery vehicle that synergistically scavenged ROS and inhibited M1 polarization while enabling the sustained release of exosomes. The exosomes further promoted M2 polarization and subsequently alleviated synovial inflammation. This synergistic strategy combining the intrinsic activity of PDA with exosome-mediated signal transduction achieved therapeutic efficacies superior to those of single-component interventions. Furthermore, integrating macrophage regulation with other therapeutic modalities has further expanded the application scope of PDA-based nanoplatforms. For example, the GHM@MFL-Gal-Mal/Cur hydrogel developed by Wang et al*.* [172] not only promoted M2 polarization but also activated the autophagy pathway of macrophages to enhance their bactericidal capacity and maintain local immune homeostasis in infected burn wounds. The correlation between autophagy activation and macrophage polarization represents an emerging mechanism whose role extends beyond simple phenotypic switching and involves fundamental cellular processes. Another form of integration combines immune regulation with remotely activatable therapeutic modalities. In the treatment of RA, Li et al*.* [131] developed a multifunctional platform (MPMAF) composed of PDA-coated MnO_2_ modified with folic acid and a TNF-α aptamer. This system integrated macrophage regulation with photothermal ablation and active targeting to address the complex pathophysiology of autoimmune diseases in a multipronged manner.

Taken together, four distinct design strategies can be distilled for PDA-based nanoplatforms in regulating macrophage polarization. These approaches encompass the direct exploitation of the intrinsic immunomodulatory activity of PDA and the amplification of this activity through combination with complementary bioactive agents such as exosomes. Furthermore, they involve the enhancement of fundamental PDA properties via compositional engineering to induce downstream effects on macrophage polarization along with the integration of macrophage regulation with alternative therapeutic modalities for multi-target interventions. Progressing from fundamental designs to sophisticated integrations, these strategies establish PDA-based nanoplatforms as versatile regulatory systems for treating a wide array of diseases associated with inflammation and tissue repair.

#### Pathway Inhibition

Signaling pathways, including STING and NF-κB, serve as critical nodes connecting oxidative stress with inflammatory responses. Excessive ROS can activate these pathways to induce the production of proinflammatory cytokines such as TNF-α, IL-1β, and IL-6, which subsequently exacerbates tissue damage and perpetuates the vicious cycle of ROS and inflammation. Targeting these pathways has emerged as an effective strategy to interrupt this feedback loop at the signaling level. PDA-based nanoplatforms participate in this strategy through multiple mechanisms where direct ROS scavenging removes upstream activation factors and engineered designs enable specific pathway inhibition or signal molecule interception.

A representative strategy entails the delivery of specific pathway inhibitors to inflammatory sites. In the treatment of RA, Shen et al*.* [173] developed polyethylenimine-coated mesoporous PDA nanoparticles loaded with the STING antagonist C-176. The PDA core played a dual role where its mesoporous structure achieved efficient loading and sustained release of C-176 and the PEI coating endowed the nanoparticles with the ability to adsorb dsDNA to reduce upstream activation of the STING pathway. This synergistic design combined direct STING inhibition with the clearance of pathway-specific stimuli to significantly downregulate the secretion of TNF-α and IL-6 and mitigate joint inflammation and damage. This example demonstrates that PDA platforms can go beyond simple drug delivery by being engineered to simultaneously intercept pathway activators and deliver inhibitors. Beyond single-pathway inhibition, researchers have explored multipronged strategies targeting multiple nodes in the signaling cascade. For the treatment of LF, Liu et al*.* [174] designed a multifunctional lysosome-targeting nanoshuttle by conjugating an ALK5-targeting ligand and a CI-M6PR ligand onto antioxidant PDA nanoparticles. This system achieved dual targeting of TGF-β receptors and hepatic stellate cells while the intrinsic antioxidant activity of PDA directly inhibited ROS-mediated TGF-β secretion. By simultaneously blocking the TGF-β/Smad2/3 signaling pathway at multiple nodes, reducing upstream ROS triggers, and targeting receptor degradation for cell-specific delivery, this approach achieved potent anti-fibrotic effects. This work indicates that pathway inhibition can be amplified through multi-point interventions rather than single-target blockade. Furthermore, combining PDA nanoplatforms with cutting-edge gene therapy can achieve the most profound pathway blockade at the post-transcriptional level. For instance, Li et al*.* [116] recently developed microRNA-146a-loaded aminated PDA composite nanoparticles for the treatment of atherosclerosis. The PDA core not only facilitated lysosomal escape of nucleic acids via the proton sponge effect and scavenged free ROS but also delivered miR-146a to specifically silence key regulatory proteins within the NF-κB pathway such as IRF5 and TRAF6. This dual strike of intrinsic antioxidation and specific gene silencing successfully stabilized atherosclerotic plaques.

Beyond the direct delivery of inhibitors and multi-point targeting, pathway inhibition can also be achieved through indirect mechanisms that modulate the inflammatory microenvironment. For example, in the treatment of myocardial infarction, Zheng et al*.* [175] discovered that a Rb1-loaded PDA hydrogel inhibited the activation of the cGAS-STING pathway and alleviated inflammatory damage by scavenging ROS. This example demonstrates that even in the absence of direct pathway inhibitors, the antioxidant function of PDA can attenuate inflammatory signaling by removing the oxidative triggers that sustain pathway activation (Fig. 4b). The applicability of pathway inhibition strategies is not limited to peripheral inflammation but can also be extended to central nervous system disorders. For instance, PDA-based nanoplatforms can indirectly inhibit the NF-κB pathway by regulating the neuron-microglia communication axis. Jiang et al*.* [176] constructed a SOD and Cu ion-coordinated PDA microgel system (mCu-PDA/SOD) for the treatment of Parkinson's disease. While scavenging ROS, the degradation of PDA released dopamine to promote neuronal repair and inhibit the release of neuronal CX3CL1. This subsequently blocked the activation of the CX3CR1 receptor-mediated NF-κB/NLRP3 pathway in microglia to reduce the release of inflammatory factors such as IL-1β and alleviate neuroinflammation. This research revealed a novel mechanism by which PDA-based nanoplatforms achieve NF-κB pathway inhibition by intervening in intercellular communication.

Synthesizing these examples allows for the extraction of three primary design strategies for PDA-based nanoplatforms in pathway inhibition. These approaches involve directly delivering specific pathway inhibitors while utilizing the multifunctional carrier properties of PDA to intercept upstream activation factors. Another strategy encompasses multi-point pathway targeting that integrates receptor degradation and upstream ROS clearance with cell-specific delivery and gene therapy to achieve specific post-transcriptional gene silencing. The final strategy entails the indirect attenuation of pathway signaling through the intrinsic ROS-scavenging activity of PDA to remove the oxidative triggers responsible for sustaining inflammatory signals. Ranging from targeted interventions to broad microenvironmental modulation, these comprehensive approaches establish PDA-based nanoplatforms as highly effective therapeutic systems for disrupting inflammatory signaling cascades.

#### cfDNA Scavenging

Derived primarily from the nuclei and mitochondria of necrotic cells, cfDNA has emerged as a crucial proinflammatory mediator in various inflammatory diseases [177, 178]. Within the pathological microenvironment, excessive cfDNA activates the TLR9/NF-κB signaling pathway to induce ROS generation and amplify inflammatory responses. Scavenging cfDNA represents an upstream intervention strategy that removes the inflammatory stimulus itself rather than merely blocking its downstream effects. PDA-based nanoplatforms are particularly well-suited for this strategy owing to their tunable surface chemistry which enables engineered designs to achieve positively charged surfaces for the electrostatic binding of cfDNA.

Modifying PDA with cationic groups is a direct way to enhance cfDNA capture efficiency. For the treatment of RA, Chen et al*.* [83] developed dimethylamine-modified PDA nanoparticles to render their surfaces positively charged. These cationic PDA nanoparticles exhibited strong binding affinity for cfDNA to effectively sequester it and prevent its interaction with TLR9 receptors. This cfDNA scavenging activity inhibited cfDNA-induced inflammation and reduced the expression of proinflammatory cytokines while mitigating joint damage in arthritis models. Importantly, this study demonstrated that surface charge engineering alone can impart therapeutic effects via cfDNA clearance to establish a foundational strategy for PDA-based nanoplatforms. More recently, cfDNA scavenging has been combined with other therapeutic functions in multifunctional platforms. In the context of diabetic wound healing, Xiao et al*.* [179] developed MPDA-PEI@GelMA hydrogel microspheres where mesoporous PDA nanoparticles were modified with cationic polyethylenimine (PEI) to enhance cfDNA capture. The MPDA core contributed its intrinsic ROS-scavenging activity while the PEI coating facilitated efficient cfDNA binding. This bifunctional design simultaneously addressed the two interconnected pathological drivers of excessive ROS and proinflammatory cfDNA to significantly reduce the secretion of TNF-α, IL-1β and IL-6 within the wound microenvironment (Fig. 4c). Combining cfDNA clearance with antioxidant activity elegantly demonstrates how multiple upstream interventions can be synergistically deployed.

These examples reveal two distinct strategies for cfDNA scavenging based on PDA platforms. The first approach relies solely on surface charge engineering to impart therapeutic capabilities for binding cfDNA through simple cationic modifications. The second strategy involves integrating cfDNA clearance with complementary functions such as ROS elimination to create versatile platforms capable of addressing interconnected pathological drivers. Both strategies leverage the adaptable surface chemistry of PDA as the foundation for cfDNA binding which successfully establishes PDA nanoplatforms as effective systems for upstream intervention in diseases driven by inflammation.

#### Gut Microbiota Modulation

The gut microbiota plays a pivotal role in maintaining intestinal homeostasis and modulating immune responses. Dysbiosis involving the perturbation of microbial composition is increasingly recognized as a critical factor in the pathogenesis of IBD and other diseases driven by inflammation [180]. The perturbed microbial community compromises intestinal barrier integrity and promotes the infiltration of proinflammatory immune cells to create a microenvironment conducive to persistent oxidative stress and inflammation. Modulating the gut microbiota represents an upstream ecological strategy that targets the environmental drivers of intestinal inflammation to complement direct ROS scavenging and immunomodulatory approaches [92, 181].

A fundamental strategy is the direct administration of PDA nanoparticles to exert a dual effect on the intestinal microenvironment. Ding et al*.* [182] developed ultrasmall PDA nanodots (PDA NDs) with an average diameter of approximately 3 nm as oral nanomedicines for targeted IBD therapy. These negatively charged PDA NDs possess broad-spectrum antioxidant activity and excellent gastrointestinal stability, enabling them to efficiently scavenge ROS while naturally accumulating at inflamed intestinal sites through electrostatic interactions. In addition to their direct antioxidant effects, the PDA NDs significantly modulated the gut microbiota composition to restore microbial homeostasis in mouse models of IBD with varying severities (Fig. 4d). This work demonstrates that PDA nanoparticles alone can simultaneously address oxidative stress and microbial dysbiosis without the need for additional bioactive payloads, thereby targeting two interconnected drivers of intestinal inflammation.

Building upon this concept, researchers have developed more sophisticated platforms that integrate microbiota modulation with enhanced targeting and delivery capabilities. Yang et al*.* [125] designed PDA-coated prebiotic yeast beta-glucan nanocomplexes for the oral treatment of IBD. The PDA coating fulfilled multiple functions where its robust mucoadhesive properties enabled firm attachment to the inflamed colonic mucosa to significantly prolong intestinal retention. Simultaneously its intrinsic ROS-scavenging activity synergistically inhibited inflammatory responses alongside the prebiotic core while its protective shell enhanced the stability of the encapsulated beta-glucan during gastrointestinal transit. The YBNs@PDA system reduced proinflammatory cytokine levels to regulate the gut microbiota composition and promote the proliferation of beneficial bacteria, thereby achieving therapeutic efficacy through orchestrated antioxidant and prebiotic effects. Furthermore, the strategy of modifying probiotics with polyphenol nanoparticles [98] provides a valuable reference for the future expansion of PDA-based nanoplatforms.

These examples demonstrate two complementary strategies for gut microbiota modulation using PDA-based nanoplatforms. The first strategy exploits the intrinsic properties of ultrasmall PDA nanoparticles through direct oral administration to achieve the dual functions of ROS scavenging and microbiota regulation. The second approach combines the mucoadhesive and antioxidant capabilities of PDA with prebiotic components to create hybrid systems that integrate microbiota modulation with targeted intestinal delivery. Both strategies recognize that intestinal inflammation originates from complex interactions between oxidative stress and microbial dysbiosis which effectively positions PDA-based nanoplatforms as ecological regulatory systems capable of addressing these interconnected pathological drivers through targeted environmental interventions.

### Overcoming Biological Barriers for Targeted Delivery

Physiological barriers represent a primary impediment to the effective treatment of oxidative stress-related diseases among which the BBB is the most formidable obstacle that prevents the majority of small-molecule drugs and nearly all macromolecular therapeutics from entering the brain parenchyma [183]. Simultaneously other biological barriers such as the intestinal epithelium and synovium and corneal epithelium also restrict the accumulation of drugs within inflamed or damaged tissues [184]. Indeed, as highlighted by recent research consensus the development of advanced delivery vehicles capable of efficiently traversing complex physiological barriers has emerged as a critical challenge for the nanomedicine field to overcome therapeutic bottlenecks for both cutting-edge nucleic acid drugs and small-molecule targeted agents [185]. PDA-based nanoplatforms with their highly customizable surface chemistry can seamlessly incorporate these advanced surface engineering strategies to exploit endogenous transport pathways and address these delivery challenges.

#### BBB Penetration via Peptide Modification

The BBB constitutes the most significant barrier to drug delivery within the central nervous system, preventing the vast majority of therapeutic agents from reaching the brain parenchyma. Peptide-mediated targeting strategies leverage receptors highly expressed on brain microvascular endothelial cells to facilitate transcytosis, which has emerged as an exceptionally effective approach to overcome this barrier [186]. PDA nanoparticles serve as ideal platforms for this strategy because their abundant surface functional groups enable the facile and stable conjugation of targeting peptides while fully preserving their biological activity.

**Fig. 5:**
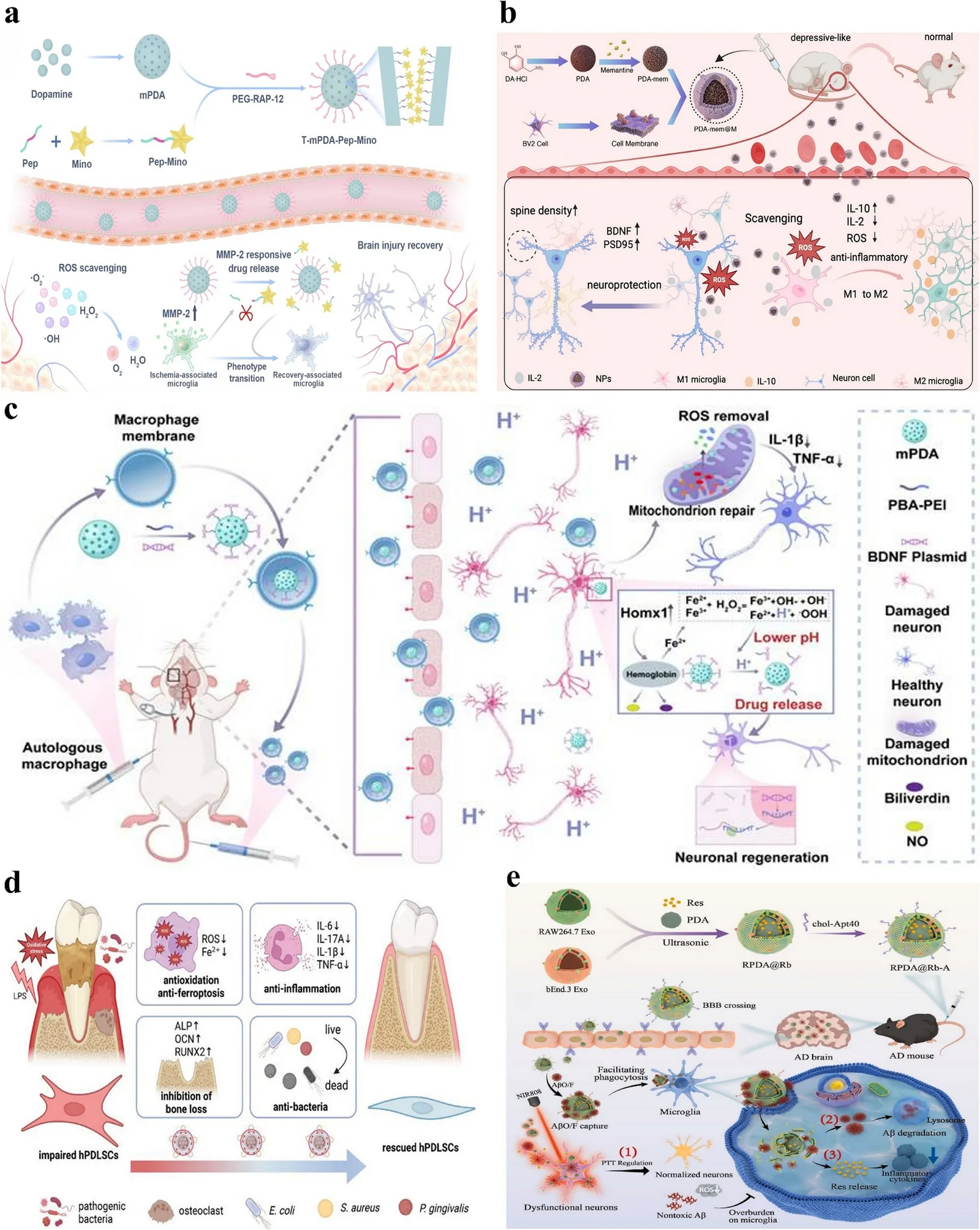
**a** RAP-12 peptide-modified MPDA nanoparticles achieve brain-targeted delivery via LRP-1-mediated transcytosis, reducing infarct volume in ischemic stroke. Reproduced with permission [103]. Copyright 2023, Springer Nature. **b** Microglia membrane-coated PDA nanoparticles loaded with memantine traverse the BBB and reverse synaptic dendritic spine loss in depression. Reproduced with permission [191]. Copyright 2025, Wiley. **c** MM-coated MPDA nanoparticles loaded with BDNF achieve pH-responsive drug release in the acidic ischemic microenvironment. Reproduced with permission [193]. Copyright 2026, Ivyspring International Publisher. **d** cRGD-modified cell membrane-coated PDA nanoparticles enhance periodontal tissue accumulation through dual-targeting. Reproduced with permission [194]. Copyright 2024, Wiley. **e** Hybrid exosome-coated PDA nanoparticles achieve BBB crossing and photothermal disaggregation of Aβ plaques in AD. Reproduced with permission [197]. Copyright 2025, Elsevier

A representative strategy involves targeting the low-density lipoprotein receptor-related protein 1 which is highly expressed on BBB endothelial cells. Wu et al*.* [103] conjugated the RAP-12 peptide onto MPDA nanoparticles via a thiol-PEG linker to construct a brain-targeting nanoplatform for the treatment of ischemic stroke. RAP-12 specifically recognized LRP-1 and triggered receptor-mediated endocytosis to achieve active transport across the BBB. Compared with the non-targeted control group, the RAP-12 modified nanoparticles exhibited a 451% increase in brain fluorescence intensity, demonstrating the profound impact of peptide modification on central nervous system drug accumulation. Beyond enhanced brain delivery, the intrinsic antioxidant activity of PDA enabled effective ROS scavenging in the ischemic brain, contributing to reduced infarct volume and improved neurological outcomes (Fig. 5a). This work reveals that even a single targeting ligand when correctly conjugated onto PDA nanoparticles can significantly enhance brain delivery.

Building upon this concept, researchers have developed peptide-modified platforms that combine BBB penetration with disease-specific targeting. For the treatment of AD, Huang et al*.* [187] modified PDA nanoparticles with the KLVFF peptide (PDA@K) which not only promoted BBB crossing but also captured disaggregated Aβ to enhance microglial uptake and degradation. Advancing this concept further Yin et al*.* [146] developed K8 peptide-modified PDA-Ru nanoparticles (K8@Fe-Rh/PDA) where the same peptide molecule was engineered to execute dual functions simultaneously. Unlike the KLVFF peptide in Huang's work, which primarily targeted Aβ with BBB penetration as a secondary benefit, the K8 peptide in this system was specifically designed to recognize BBB receptors and bind Aβ aggregates simultaneously. This integrated design achieves a higher level of molecular economy as a single peptide sequence encodes two targeting instructions to potentially realize stronger synergistic efficacy through coordinated actions on both the barrier and the disease target. The versatility of peptide modification is also reflected in the ability to select different targeting targets according to specific disease contexts. For instance, in PD treatment Lei et al*.* [188] utilized RVG29 peptide-modified PDA-curcumin nanoparticles (RPC) to achieve efficient BBB penetration and subsequent regulation of α-synuclein aggregation.

Synthesizing these examples reveals several fundamental design principles for PDA-based nanoplatforms to achieve peptide-mediated BBB penetration. The targeting peptides must be stably conjugated while fully preserving their receptor binding activity which is readily facilitated by the versatile surface chemistry of PDA. Furthermore, the peptide modification alone is sufficient to significantly enhance brain accumulation. Peptides can also be sophisticatedly engineered to seamlessly integrate the dual functionalities of barrier penetration and disease-specific targeting. These comprehensive principles establish peptide-modified PDA nanoparticles as universally applicable targeted delivery systems for central nervous system therapeutics.

#### Biomimetic Camouflage with Cell Membranes

The biomimetic camouflage of cell membrane coating has emerged as a transformative strategy for targeted drug delivery. By wrapping synthetic nanoparticles with naturally derived cell membranes, these platforms inherit the surface properties of their parent cells to achieve immune evasion, prolonged circulation time and active homing toward inflamed or diseased tissues [189, 190]. PDA nanoparticles represent the ideal core for this strategy because they provide a structurally rigid and chemically versatile scaffold capable of both stably supporting the membrane coating and contributing intrinsic antioxidant and photothermal functionalities.

MM coating represents one of the most extensively investigated camouflage strategies by capitalizing on the natural homing capability of macrophages toward inflammatory sites. For the treatment of IBD, Bao et al*.* [92] developed MM-coated PDA nanoparticles conjugated with the antimicrobial peptide mCRAMP (MMs/PDA/mCRAMP). The MM coating endowed the nanoparticles with inflammation-targeting capabilities via embedded chemokine receptors such as CCR2 and CXCR1 to preferentially accumulate at inflamed intestinal tissues. Simultaneously, the PDA core contributed ROS scavenging and immunomodulatory activities to form a synergistic platform combining active targeting with therapeutic functions. This work demonstrates that cell membrane coating can transform PDA nanoparticles from passive carriers into actively homing therapeutic systems. Building on this concept, researchers have further explored diverse membrane sources tailored to specific disease contexts. Addressing central nervous system disorders, Jiang et al*.* [191] designed microglia membrane-coated memantine-loaded PDA nanoparticles (PDA-Mem@M) for depression therapy. Microglia serving as resident immune cells of the brain possess membranes with an inherent tropism toward neuroinflammatory lesions. This system exploited the homotypic targeting capability of the microglial membrane to successfully traverse the BBB and accumulate in inflamed brain regions. The PDA core scavenged excessive ROS to alleviate the neuroinflammatory microenvironment. Concurrently the loaded memantine was released in a pH-responsive manner via Schiff base reactions to upregulate brain-derived neurotrophic factor expression and reverse the loss of synaptic dendritic spines (Fig. 5b). Furthermore, mesenchymal stem cell membrane-coated MPDA (MPDA@MSC) has also been applied in PD treatment [192] to achieve BBB crossing and inflammatory homing via VLA-4 and VCAM-1 interactions, thereby expanding the repertoire of membrane sources.

The versatility of membrane camouflage also extends to acute inflammatory diseases. In the treatment of ischemic stroke Zheng et al*.* [193] developed MM-coated MPDA nanoparticles loaded with brain-derived neurotrophic factor (MPP-B@MM). The activated MM coating enabled targeted delivery toward inflamed brain tissues while the PDA core provided pH-responsive drug release within the acidic ischemic microenvironment (Fig. 5c). For ALI therapy Zhao et al*.* [91] designed MM-coated MPDA nanoparticles loaded with peimine (MM@MPDA-PM) to achieve targeted accumulation within inflamed lung tissues while effectively regulating neutrophil extracellular traps and macrophage polarization. Beyond single-membrane coatings researchers have also engineered hybrid membrane platforms that combine the advantages of multiple cell types. In periodontitis treatment, Pan et al*.* [194] designed cRGD-modified cell membrane-coated PDA nanoparticles loaded with minocycline (PM@RCM). The cRGD modification incorporated active targeting capabilities onto the intrinsic homing properties of the cell membrane to create a dual-targeting system with enhanced accumulation in periodontal tissues (Fig. 5d).

The aforementioned examples demonstrate the versatility and profound potential of biomimetic camouflage in the targeted delivery of PDA-based nanoplatforms where diverse membrane sources provide distinct advantages. When coupled with the intrinsic therapeutic functions of PDA and its capacity to serve as a stable core for membrane coating, these biomimetic platforms represent a powerful approach to overcoming biological barriers and achieving targeted delivery to various diseased sites.

#### Exosome-Mediated Transcytosis

As endogenous nanovesicles, exosomes possess inherent capabilities for intercellular communication and crossing biological barriers which renders them attractive drug delivery vehicles. Their natural origin endows them with low immunogenicity and excellent biocompatibility along with targeting capabilities facilitated by surface adhesion molecules [195]. Nevertheless, limitations such as restricted drug loading and rapid in vivo clearance have prompted researchers to develop hybrid platforms that integrate exosomes with synthetic nanoparticles [196]. PDA nanoparticles serve as ideal cores for such composite systems because they offer high drug-loading capacities and stimuli-responsive release alongside intrinsic antioxidant functions while fully preserving the biological activity of the exosome coating.

A representative work for this strategy is the hybrid exosome-coated PDA nanoparticle (RPDA@Rb-A) constructed by Du et al*.* [197] for the treatment of AD. They fused the membranes of brain microvascular endothelial cell-derived exosomes and macrophage-derived exosomes and subsequently combined this hybrid membrane with resveratrol-loaded PDA nanoparticles and an Aβ-targeting aptamer. The resulting platform leveraged the complementary advantages of both exosome sources where the endothelial exosome component promoted BBB penetration via receptor-mediated transcytosis while the macrophage exosome component provided inflammation homing capabilities. This dual-source design achieved efficient BBB crossing and selective accumulation at Aβ plaques. Concurrently under NIR irradiation PDA mediated the photothermal disaggregation of Aβ assemblies while its intrinsic ROS-scavenging activity alleviated oxidative stress within the pathological microenvironment (Fig. 5e). This work illustrates how exosome-PDA composites synergistically overcome biological barriers and achieve multimodal therapeutic efficacies. Beyond this example, other studies have explored exosome-PDA composite platforms in diverse disease contexts such as OA [171] and LF [198], where the exosome coating enhanced targeting capabilities while PDA contributed antioxidant and immunomodulatory functions. These platforms collectively demonstrate the advantages of integrating the biological targeting capacity of exosomes with the physicochemical versatility of PDA.

In summary, transcytosis mediated by exosomes serves as a potent strategy to overcome biological barriers. PDA nanoparticles function as ideal scaffolds for exosome coatings not only to achieve stable integration and high drug-loading capacities but also to contribute supplementary therapeutic functionalities. More importantly, the modular nature of this approach enables precise adaptation to the pathological features of diverse diseases through the selection of exosomes from distinct sources and the customization of the physicochemical properties of the PDA core. This versatility successfully establishes the composite of exosomes and PDA as a targeted delivery platform with broad applicability.

### Remodeling the Regenerative Microenvironment

Tissue repair following injury or degeneration requires an orchestrated regenerative microenvironment characterized by adequate vascularization, a supportive extracellular matrix (ECM), and a balanced immune milieu. In pathological conditions such as chronic wounds, spinal cord injuries, and myocardial infarction, oxidative stress disrupts this delicate balance by impairing angiogenesis and promoting excessive inflammation, which ultimately compromises tissue regeneration [199, 200]. PDA-based nanoplatforms participate in microenvironmental remodeling through two complementary mechanisms by promoting angiogenesis to restore blood supply and by providing structural scaffolds that guide tissue formation while protecting therapeutic cells from oxidative damage. This section will explore these two design strategies for promoting tissue repair.

#### Promoting Angiogenesis

Angiogenesis is crucial for delivering oxygen and nutrients to regenerating tissues. In oxidative stress-mediated injuries, excessive ROS impair endothelial cell function and disrupt proangiogenic signaling to delay neovascularization, thereby hindering tissue repair [201]. PDA-based nanoplatforms address this challenge through multiple mechanisms where their antioxidant activity protects endothelial cells from oxidative damage while their drug delivery capabilities enable the controlled release of proangiogenic factors. Furthermore, the photothermal properties of PDA can stimulate angiogenesis through mild heating.

Using PDA to deliver proangiogenic agents is a widely adopted strategy. For diabetic wound healing Luo et al*.* [202] dispersed MPDA nanoparticles loaded with DFO into a gelatin methacryloyl hydrogel. Deferoxamine (DFO) is a recognized hypoxia mimetic agent that stabilizes HIF1α and promotes vascular endothelial growth factor (VEGF) expression. The PDA core exerted dual functions where its abundant quinone groups formed pH-responsive imine bonds with the amino groups of DFO to achieve sustained release within the acidic diabetic wound microenvironment, while its intrinsic ROS-scavenging activity alleviated oxidative stress. This approach of combining proangiogenic drug delivery with the amelioration of the oxidative microenvironment significantly enhanced neovascularization and accelerated wound healing.

Building upon this concept, researchers have further explored synergistic combinations to amplify angiogenic effects. In burn wound healing, Xue et al*.* [152] developed a composite aerogel incorporating reduced graphene oxide and PDA and silver nanoparticles along with cotton gauze. PDA promoted the structural stability of the aerogel through the formation of carbon nitrogen bonds while simultaneously scavenging ROS and inhibiting inflammatory cells. This system upregulated TGF-β1 and promoted VEGF secretion to achieve enhanced angiogenesis through orchestrated antioxidant and anti-inflammatory and proangiogenic activities. Notably, when addressing such complex wounds accompanied by severe pathogen infections, the robust interfacial crosslinking capability of PDA can even be utilized to bypass polymer matrices and directly assist in the bottom-up assembly of ultrasmall silver nanoclusters into macroscopic antibacterial aerogels. This achieves potent pathogen clearance, which is a critical prerequisite for subsequent tissue microenvironment remodeling and angiogenesis in wound-healing contexts [203].

**Fig. 6:**
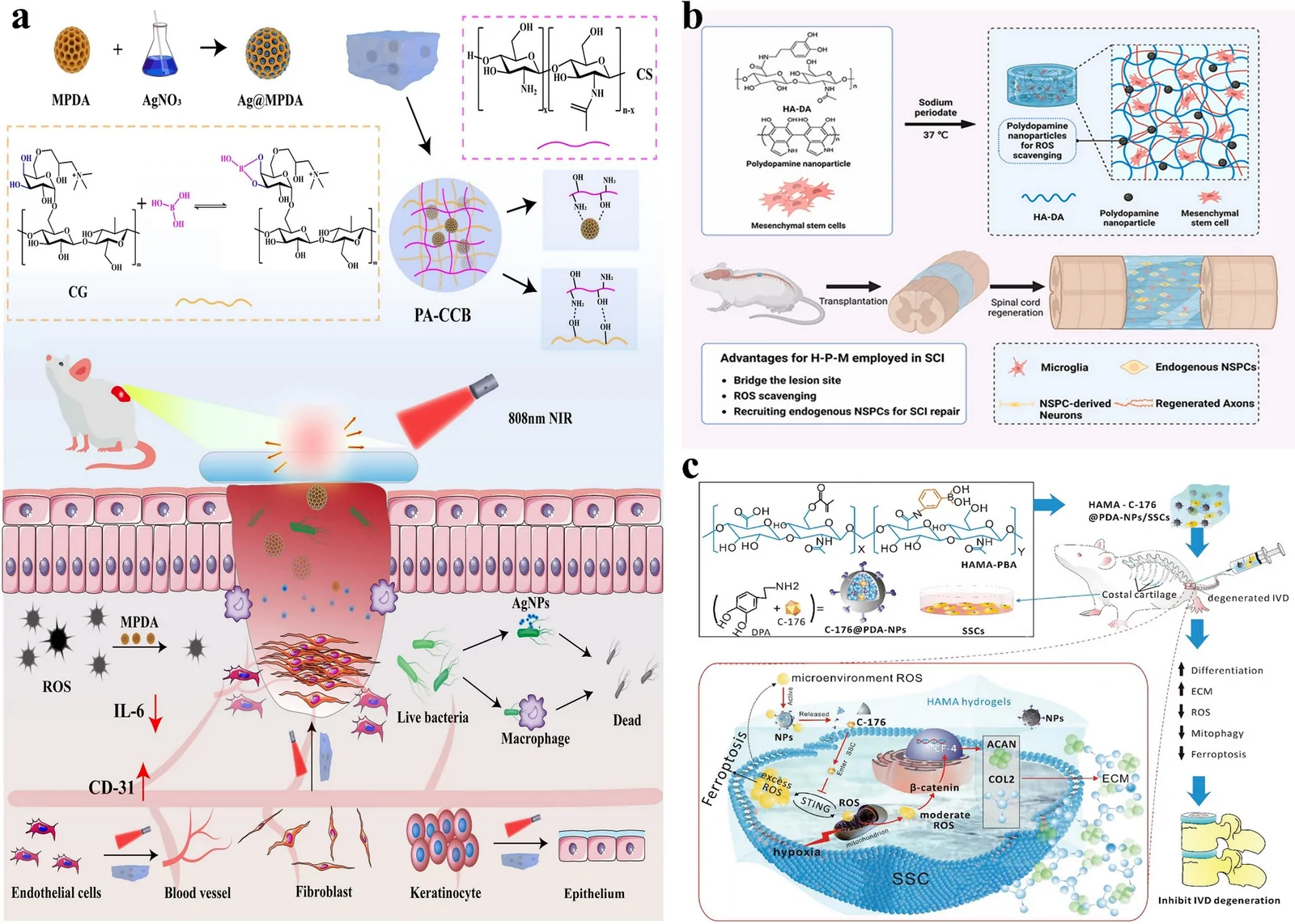
**a** Ag@MPDA nanoparticle-incorporated hydrogel promotes angiogenesis through photothermal stimulation, enhancing diabetic wound healing. Reproduced with permission [204]. Copyright 2025, Elsevier. **b** HA-conjugated hydrogel integrated with PDA nanoparticles and mesenchymal stem cells reduces ROS levels and enhances neural repair in SCI. Reproduced with permission [208]. Copyright 2025, KeAi. **c** ROS-responsive HAMA hydrogel scaffold loaded with C-176@PDA nanoparticles releases payload upon ROS trigger, inhibiting mitophagy-mediated ferroptosis and promoting stem cell differentiation in intervertebral disc degeneration. Reproduced with permission [209]. Copyright 2025, Wiley

The versatility of PDA-mediated angiogenesis also extends to physical stimulation methods. In diabetic wound healing, He et al*.* [204] developed a PA-CCB hydrogel incorporating Ag@MPDA nanoparticles. Beyond the proangiogenic effects of silver nanoparticles and the antioxidant activity of PDA, this system utilized the photothermal conversion capability of PDA to generate mild heat under NIR irradiation. This photothermal stimulation enhanced local microcirculation and activated cellular viability while synergizing with the biochemical signals of the materials to promote angiogenesis (Fig. 6a). This work demonstrates that the physicochemical properties of PDA can be actively harnessed to stimulate vascularization through physical mechanisms that complement biochemical delivery. Besides single-drug delivery, combination delivery strategies have also shown particular promise. For diabetic wound healing, Dai et al*.* [149] developed the CPO/D@P/IGF-1 hydrogel where PDA nanoparticles adsorbed DFO on their surfaces to achieve sustained release while the hydrogel matrix incorporated IGF-1. The photothermal conversion of PDA was utilized to promote M2 macrophage polarization and enhance angiogenesis, while the dual delivery of DFO and IGF-1 provided complementary proangiogenic signals. This multipronged approach achieved superior wound healing outcomes by simultaneously addressing oxidative stress and inflammation and deficient vascularization.

Synthesizing these examples reveals three distinct strategies for PDA-based nanoplatforms to promote angiogenesis. These approaches involve the controlled release of proangiogenic agents by leveraging the drug-loading capacity and microenvironment-responsive release properties of PDA. Furthermore, they integrate complementary materials or agents to amplify angiogenic signals through synergistic mechanisms. Finally, they harness the photothermal properties of PDA to stimulate angiogenesis via physical effects. These strategies are frequently combined within a single platform to establish PDA-based nanoplatforms as highly efficient pro-regenerative systems for restoring vascularization in ischemic and damaged tissues.

#### Providing Structural Scaffolds

Three-dimensional scaffolds provide structural support for cell adhesion, proliferation, and migration while protecting therapeutic cells from harsh oxidative environments [205–207]. PDA contributes to scaffold-based strategies through multiple mechanisms where its robust adhesive properties enable stable integration with various scaffold materials and its intrinsic antioxidant activity creates a protective microenvironment for encapsulated cells while its versatile surface chemistry allows for the functional modification of bioactive molecules. This section will explore how PDA-based nanoplatforms utilize structural scaffolds to enhance tissue regeneration.

A representative strategy involves integrating PDA nanoparticles into hydrogel scaffolds to create a cytoprotective environment for stem cell transplantation. For the treatment of SCI, Kao et al*.* [208] developed a composite hydrogel comprising a HA-conjugated hydrogel and PDA nanoparticles combined with human bone marrow mesenchymal stem cells (H-P-M hydrogel). The PDA nanoparticles exerted dual functions within the scaffold where they reduced intracellular ROS levels in microglia by 65% to alleviate oxidative stress at the lesion site while their incorporation into the HA hydrogel enhanced the mechanical stability and tissue adhesiveness of the scaffold. This cytoprotective effect enabled the transplanted stem cells to survive and function within the oxidative spinal cord microenvironment thereby promoting neural repair through synergistic antioxidant support and structural guidance (Fig. 6b). Combining PDA scaffolds with smart responsiveness and deep mechanistic regulation further elevated the efficacy of tissue regeneration. Luo et al*.* [209] developed a ROS-responsive HAMA hydrogel scaffold loaded with C-176@PDA nanoparticles and co-cultured with skeletal stem cells for the treatment of intervertebral disc degeneration. This scaffold crosslinked HAMA with C-176@PDA via 3-aminophenylboronic acid to form ROS-responsive boronate ester bonds. Within the hypoxic microenvironment of intervertebral disc degeneration, excessive ROS triggered the cleavage of these boronate ester bonds to release the C-176@PDA nanoparticles. The released C-176 inhibited the cGAS/STING pathway to reduce ROS accumulation within the stem cells and subsequently suppressed mitophagy-mediated ferroptosis via the HIF-1α/FOXO3 axis. Concurrently, the moderate ROS levels activated the Wnt/β-catenin signaling pathway to promote the differentiation of skeletal stem cells into nucleus pulposus-like cells and effectively delay the progression of intervertebral disc degeneration (Fig. 6c).

The versatility of PDA-based scaffolds also extends to applications that require localized and long-term drug retention. In dynamic and complex in vivo microenvironments such as spinal cord injuries the stable fixation of implants and the sustained release of drugs frequently confront immense challenges. Recently, Li et al*.* [210] developed an injectable short nanofiber composite scaffold modified with PDA (ISN@n-BAK) to perfectly address this delivery challenge. Within this system, the PDA coating on the surface of the electrospun short nanofibers played an exceedingly critical dual role. Primarily, its exceptional intrinsic tissue adhesiveness enabled the injected fibrous scaffold to firmly anchor at the SCI lesion, thereby preventing the displacement or loss of the material. Furthermore, the catechol groups on the PDA surface served as highly active reactive interfaces to efficiently graft and immobilize ROS-responsive liposomes encapsulating DNA protective agents (n-BAK). This synergistic strategy based on the in situ adhesion of PDA and stable chemical grafting successfully established a long-acting microenvironmental regulatory reservoir locally at the injury site. It not only provided biomimetic guidance for neurite extension but also continuously quenched excessive ROS at the biochemical level while releasing targeted drugs on demand to successfully reverse the trajectory of oxidative stress-induced cellular senescence. This work comprehensively illustrates how the intrinsic adhesiveness and interfacial reactivity of PDA can transform a mere physical scaffold into a robust structural platform capable of achieving long-term local retention and sustained biochemical intervention.

Synthesizing these examples reveals three distinct strategies by which PDA-based nanoplatforms utilize structural scaffolds for tissue regeneration. The first approach involves directly incorporating PDA into hydrogels to create a cytoprotective microenvironment for transplanted cells. The second strategy integrates drug-loaded PDA nanoparticles into scaffolds to seamlessly combine structural support with localized therapy. The third method designs PDA-based reservoir systems to establish a sustained biochemical microenvironment through prolonged retention. These integrated strategies elegantly transform scaffolds from passive physical supports into multifunctional platforms that actively participate in tissue regeneration through antioxidant protection and immunomodulation and controlled drug delivery. Furthermore, this paradigm highly aligns with the latest development trends of smart hydrogel-based delivery platforms in the field of bone and soft tissue regeneration to highlight the profound integration of microenvironmental responsiveness and biological activity [211].

### Regulating Programmed Cell Death Pathways

Beyond necrosis oxidative stress can trigger specific programmed cell death pathways that include ferroptosis and pyroptosis as well as apoptosis. These pathways amplify tissue damage through distinct pathological mechanisms [212]. Ferroptosis involves iron-dependent lipid peroxidation [213], while pyroptosis is driven by inflammasome activation [214] and apoptosis proceeds through mitochondrial signaling cascades [215]. PDA intervenes in these interconnected pathways through its iron-chelating capacity and ROS-scavenging activity along with its mitochondrial protective effects. This section explores how PDA-based nanoplatforms can be rationally designed to modulate these intricate programmed cell death pathways.

#### Ferroptosis Inhibition

Ferroptosis represents a recently identified form of programmed cell death driven by iron-dependent lipid peroxidation and has emerged as a critical pathological mechanism in a variety of oxidative stress-related diseases [134]. In recent years, multidimensional engineered interventions based on nanomaterials have demonstrated immense therapeutic potential for the precise regulation of ferroptosis [216]. PDA exerts an intrinsic inhibitory effect on ferroptosis through multiple mechanisms where its catechol groups primarily chelate free Fe^2^⁺ to suppress the Fenton reaction and the subsequent generation of hydroxyl radicals. Furthermore, PDA is capable of preserving the activity of glutathione peroxidase 4 (GPX4) by inhibiting its ubiquitin-mediated degradation. This section explores the rational design of PDA-based nanoplatforms to harness these distinctive properties for effective ferroptosis inhibition.

**Fig. 7:**
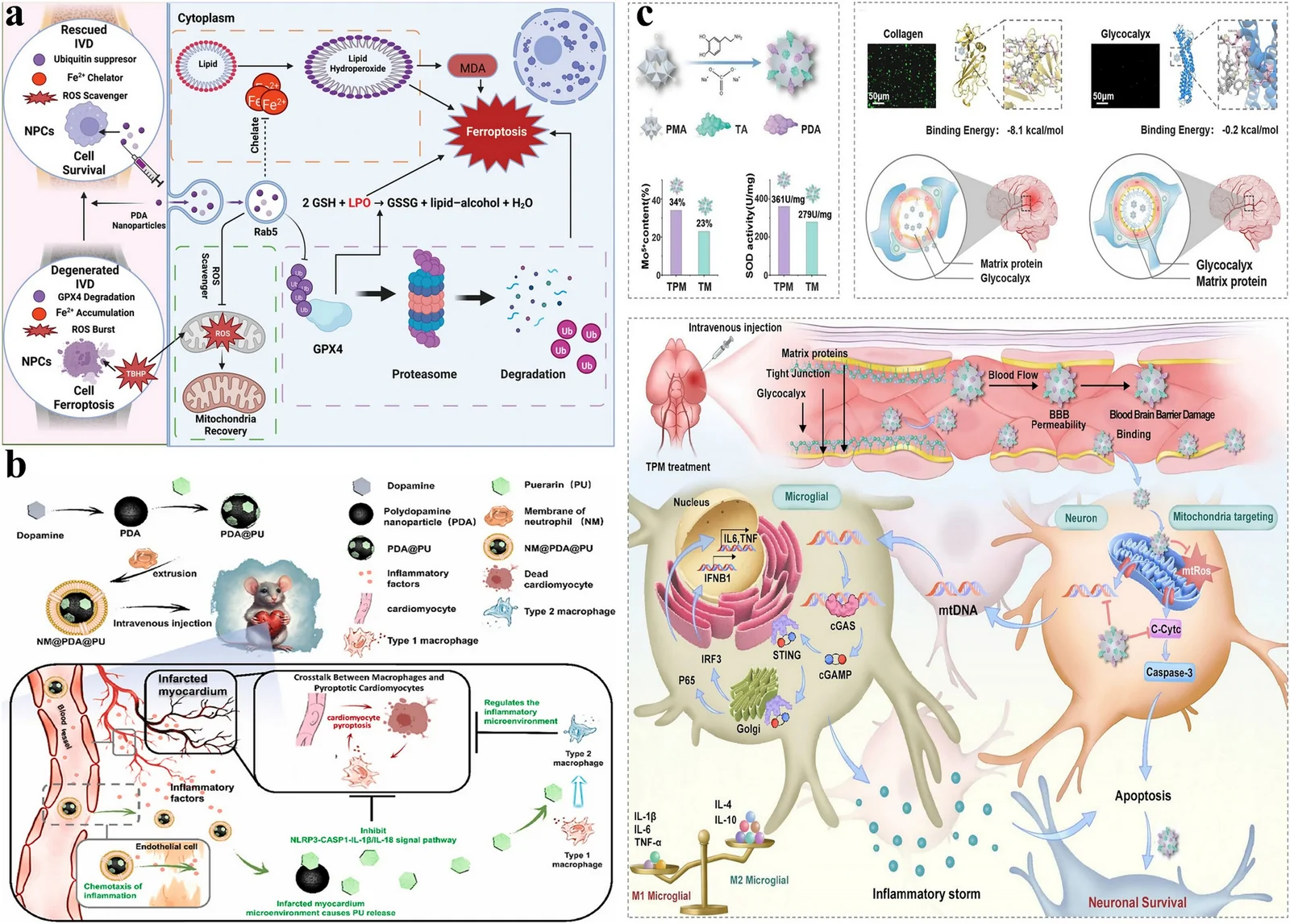
**a** PDA nanoparticles inhibit ferroptosis in nucleus pulposus cells by chelating Fe^2^⁺, regulating iron storage protein expression, and suppressing GPX4 ubiquitination. Reproduced with permission [218]. Copyright 2023, Wiley. **b** Neutrophil membrane-coated PDA nanoparticles loaded with puerarin scavenge ROS and inhibit pyroptosis in myocardial infarction. Reproduced with permission [221]. Copyright 2024, Elsevier. **c** Tannic acid-PDA-molybdenum-based ternary nanomedicine (TPM) localizes to neuronal mitochondria, scavenges mitochondrial ROS, and blocks ER stress-mediated apoptotic pathways in ischemic stroke reperfusion injury. Reproduced with permission [227]. Copyright 2025, Wiley

A direct strategy is to utilize the iron-chelating ability of PDA to capture labile iron. In the treatment of myocardial ischemia–reperfusion injury Zhu et al*.* [217] utilized MPDA as a carrier loaded with CeO_2_ nanozymes and the iron chelator dexrazoxane (DXZ) to construct a hierarchical targeted delivery system (D/Ce@MPDA-C/P). PDA not only directly scavenged ROS but also achieved the sustained release of DXZ for continuous Fe^2^⁺ chelation to effectively inhibit ferroptosis in damaged cardiomyocytes. This example demonstrates that combining the intrinsic chelating capacity of PDA with additional iron-binding agents can amplify the ferroptosis-inhibitory effect. In addition to iron chelation, PDA has also been shown to protect GPX4 from degradation. GPX4 serves as a key enzyme for scavenging lipid peroxides. In the treatment of intervertebral disc degeneration, Yang et al*.* [218] demonstrated that PDA nanoparticles effectively inhibited oxidative stress-mediated ferroptosis in nucleus pulposus cells by chelating Fe^2^⁺, regulating iron storage protein expression, co-localizing with GPX4 around mitochondria, and suppressing its ubiquitination-mediated degradation. This multipronged effect simultaneously reduced iron availability and preserved GPX4 function to achieve robust protection against ferroptosis (Fig. 7a). In addition to direct iron chelation and GPX4 preservation, PDA-based nanoplatforms can synergistically upregulate endogenous antioxidant defenses. In the treatment of atherosclerosis Dai et al*.* [219] developed FPLG nanoparticles where PDA reduced the Fenton reaction through Fe^2^⁺ chelation while synergistically upregulating GPX4 and Nrf2 activities to restore endogenous antioxidant defenses and thereby inhibit foam cell ferroptosis. This approach illustrates that PDA can serve as a foundation for multi-target interventions to simultaneously address the iron-dependent and oxidative execution phases of ferroptosis.

Synthesizing these examples reveals three distinct design strategies for PDA-based nanoplatforms to inhibit ferroptosis. These strategies include leveraging the intrinsic iron-chelating capability of PDA alone or in combination with additional chelators, preserving GPX4 activity through co-localization and the suppression of its degradation, and synergistically upregulating endogenous antioxidant pathways such as Nrf2. By targeting multiple nodes within the ferroptotic cascade, PDA-based nanoplatforms provide a versatile tool for combating iron-dependent cell death across various pathological conditions.

#### Pyroptosis Suppression

Pyroptosis is a pro-inflammatory form of programmed cell death characterized by inflammasome activation, caspase-1 cleavage, and the subsequent release of pro-inflammatory cytokines such as IL-1β and IL-18. Unlike immunologically silent apoptosis, pyroptosis amplifies the inflammatory cascade to exacerbate tissue damage in conditions such as myocardial infarction, ALI, and neurodegenerative diseases [220]. Oxidative stress serves as a crucial trigger for pyroptosis by activating the NLRP3 inflammasome. PDA primarily intervenes in this pathway through its ROS-scavenging activity to remove the oxidative triggers that initiate inflammasome assembly and thereby inhibit downstream pyroptotic cell death.

A representative example comes from the treatment of myocardial infarction where Wang et al*.* [221] designed neutrophil membrane-coated PDA nanoparticles loaded with puerarin (NM@PDA@PU). The neutrophil membrane coating enabled targeted delivery to the inflamed myocardium while the PDA core contributed intrinsic antioxidant activity that synergized with puerarin to scavenge ROS. This ROS-scavenging effect disrupted the interplay between macrophages and cardiomyocyte pyroptosis. By reducing oxidative stress within the infarcted microenvironment, this platform attenuated inflammasome activation and subsequent pyroptotic cell death (Fig. 7b). This study demonstrates that PDA-mediated ROS scavenging can indirectly inhibit pyroptosis by removing upstream oxidative triggers. Although inhibiting pyroptosis via antioxidant mechanisms remains the primary strategy in current fundamental PDA research, the aforementioned example illustrates a key principle that by mitigating oxidative stress PDA-based nanoplatforms can interrupt the inflammation-amplifying loop driven by pyroptotic cell death to offer therapeutic potential for diseases where pyroptosis plays a central pathological role.

Notably, in severe systemic inflammatory diseases such as sepsis a single-cell death pathway is often insufficient to fully encapsulate the extent of cellular damage since excessive ROS not only trigger pyroptosis but concurrently induce mitochondrial apoptosis [222]. This necessitates the development of nanoplatforms capable of simultaneously intervening in multiple interconnected death pathways [223]. Recently Yan et al*.* [224] developed an acid-responsive hollow mesoporous PDA nanoplatform targeting the inflammatory microenvironment (HMPDA@BA/NAD + @LSA) to synergistically block macrophage pyroptosis and mitochondrial apoptosis in LPS-induced sepsis. The PDA core played an indispensable foundational role within this system where the slightly acidic inflammatory lesions triggered the disassembly of HMPDA to release the loaded calcium chelator (BA-AM) and energy supplement (NAD +). Concurrently the disassembled PDA fully exposed its phenolic hydroxyl groups to act as a primary antioxidant barrier for the rapid quenching of the intracellular ROS burst. By precisely removing this critical oxidative trigger alongside the signal regulatory effects of NAD + the platform potently inhibited the assembly and activation of the NF-κB-NLRP3-ASC-Caspase-1 pyroptosis axis at its source. This prevented the formation of a pro-inflammatory cytokine storm and subsequently truncated the Bax-Cyt-c-Caspase-3 mitochondrial apoptotic cascade. This work perfectly demonstrates how the intrinsic ROS-scavenging capability and pathology-responsive structural features of PDA can be harnessed to construct a multidimensional cell death regulatory network capable of simultaneously severing the crosstalk between pyroptosis and apoptosis.

In summary, PDA functions as a key regulator of pyroptosis by primarily eliminating the ROS that initiate inflammasome assembly. Its versatile structural features further allow for the integration of synergistic payloads to potently attenuate the caspase-1 signaling axis and the subsequent release of pro-inflammatory cytokines. Consequently, the capability of PDA to address multiple interconnected cell death pathways simultaneously establishes it as a robust and multidimensional platform for mitigating tissue damage in diverse inflammatory pathological environments.

#### Apoptosis Attenuation

Apoptosis is a programmed cell death pathway characterized by caspase activation and systematic cellular dismantling to play a dual role in physiology and disease [225]. Although apoptosis is crucial for normal tissue homeostasis, excessive apoptosis under pathological oxidative stress leads to irreversible cell loss in conditions such as myocardial infarction, ischemia–reperfusion injury, and neurodegenerative diseases. Unlike inflammation-amplifying ferroptosis and pyroptosis, apoptosis is typically immunologically silent but its destructive impact remains profound when it eliminates non-renewable cells such as cardiomyocytes and neurons. PDA primarily exerts its anti-apoptotic effects through mitochondrial protection and the activation of survival pathways.

A representative strategy involves preserving mitochondrial integrity to prevent cytochrome c release and subsequent caspase activation. In the treatment of myocardial infarction, Wang et al*.* [226] developed epigallocatechin gallate (EGCG)-loaded MPDA nanoparticles embedded in a chitosan hydrogel (EGCG@MPDA/CS) where the anti-apoptotic mechanism is particularly noteworthy. The ROS-scavenging activity of PDA protected the mitochondrial membrane potential while the sustained release of EGCG activated the Nrf2/HO-1 pathway to jointly inhibit cardiomyocyte apoptosis. This dual action combining direct mitochondrial protection with the upregulation of endogenous antioxidant defenses achieved robust suppression of apoptotic cell death in the infarcted myocardium. Building upon this concept, researchers have further designed platforms that utilize the intrinsic antioxidant capacity of PDA to sever organelle crosstalk at the subcellular level and thereby intervene in the apoptotic cascade. For instance, in the treatment of ischemic stroke reperfusion injury, Shi et al*.* [227] developed a biomimetic ternary nanomedicine (TPM) composed of tannic acid, PDA, and molybdenum-based heteropolyacids. The incorporation of PDA endowed this system with exceptional ROS-scavenging capabilities. After precisely localizing to neuronal mitochondria, TPM efficiently quenched mitochondrial ROS to not only directly maintain mitochondrial integrity and prevent cytochrome c-mediated intrinsic apoptosis but also fundamentally block oxidative stress-induced endoplasmic reticulum stress and significantly downregulate key apoptosis-triggering pathways such as PERK, IRE1-α, and ATF6 (Fig. 7c). This example demonstrates that even without loading additional pro-survival drugs, a PDA-based system can break the nexus between mitochondrial damage and endoplasmic reticulum stress relying solely on highly specific subcellular targeting and potent intrinsic ROS-scavenging activity.

These examples reveal two complementary strategies for achieving apoptosis attenuation via PDA-based nanoplatforms that include activating pro-survival pathways such as Nrf2/HO-1 through controlled drug release and utilizing the intrinsic properties of PDA to directly protect mitochondria and block ER stress-mediated apoptotic networks. By comprehensively preserving cell viability under oxidative challenges these approaches complement the pyroptosis and ferroptosis regulation strategies discussed in this section to collectively construct a robust cytoprotective barrier.

### Material-Intrinsic Therapeutic Engineering

Beyond biological targeting and microenvironment regulation the inherent physicochemical properties of PDA itself provide direct therapeutic functions without relying on complex external modifications or drug loading. This material-intrinsic therapeutic engineering encompasses tunable particle size, inherent interfacial adhesiveness, responsive behavior to microenvironmental signals, and intrinsic photophysicochemical properties to enable PDA-based nanoplatforms to achieve precise intervention in the disease microenvironment purely through the material itself.

#### Size-Dependent Passive Accumulation

Nanoparticle size is a critical factor determining in vivo biodistribution and influencing circulation time, extravasation, and organ-specific accumulation [228]. Unlike active targeting strategies that require surface modification with biological ligands, size-dependent passive accumulation exploits the inherent physiological characteristics of diseased tissues. PDA nanoparticles achieve tunable size control through simple adjustments of synthetic parameters to enable their rational design for passive targeting to specific organs without the need for complex functionalization.

**Fig. 8:**
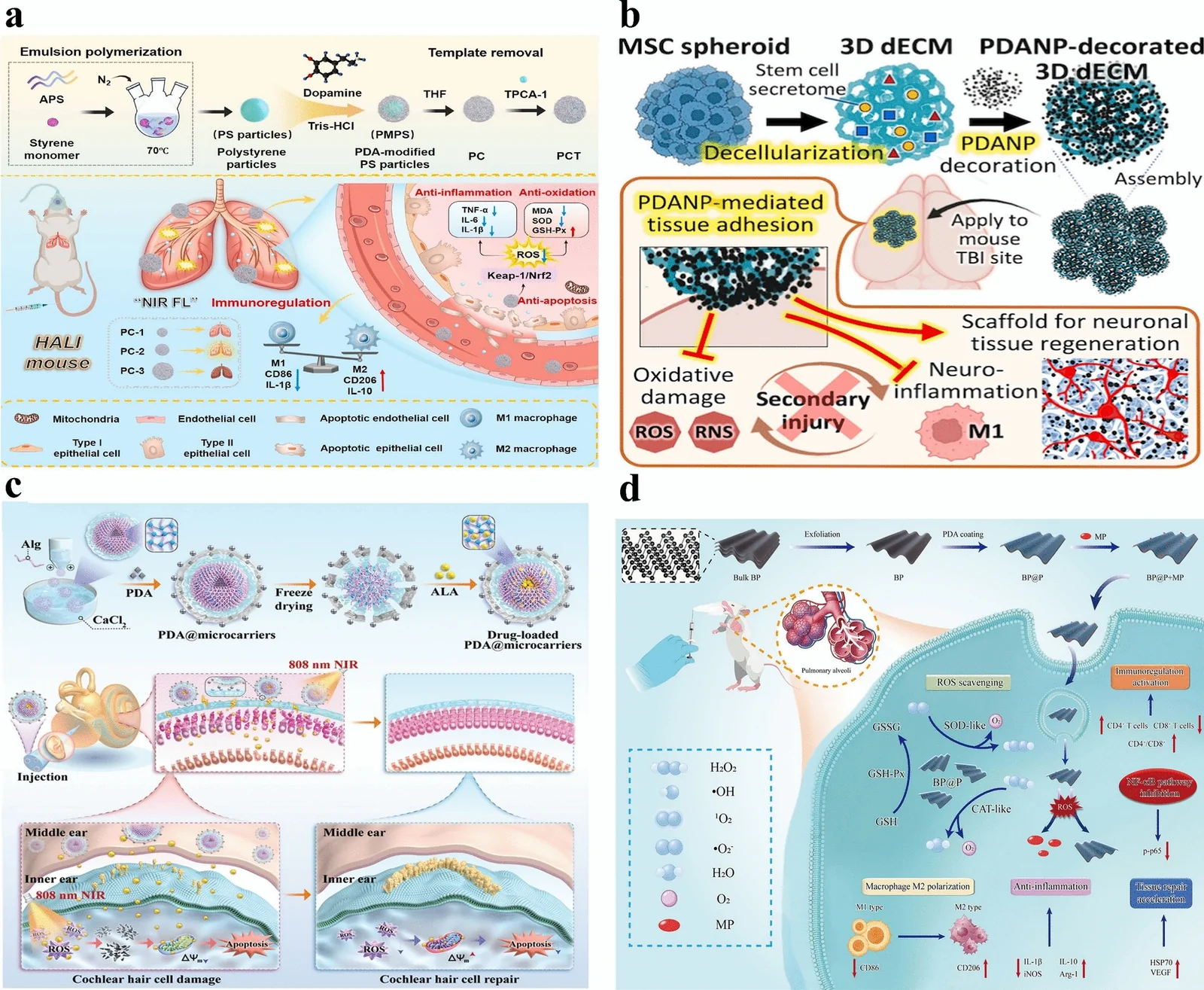
**a** Size-dependent pulmonary accumulation of PDA nanocapsules (~ 680 nm) enables efficient delivery of IKK-2 inhibitor TPCA-1 to inflamed lung tissues. Reproduced with permission [229]. Copyright 2025, Elsevier. **b** PDA nanoparticle-decorated 3D decellularized ECM scaffolds exhibit tissue adhesiveness and anchor to brain tissue surface for TBI repair. Reproduced with permission [233]. Copyright 2025, Elsevier. **c** PDA-coated alginate microcarriers achieve NIR-triggered release of α-lipoic acid for sensorineural hearing loss treatment. Reproduced with permission [239]. Copyright 2025, Wiley. **d** PDA-coated black phosphorus nanocarrier enables ROS-responsive release of methylprednisolone for ALI therapy. Reproduced with permission [240]. Copyright 2025, Elsevier

For example, Han et al*.* [229] systematically controlled the size of melanin-like PDA nanocapsules by adjusting the doping amount of dopamine precursors to generate particles ranging from 100 nm to nearly 1000 nm in diameter. By systematically evaluating pulmonary accumulation following intravenous administration, they discovered that nanocapsules with a diameter of approximately 680 nm exhibited the highest deposition in lung tissues. This size-dependent lung-targeting capability enabled the highly efficient delivery of the IKK-2 inhibitor TPCA-1 to inflamed pulmonary sites while PDA contributed additional ROS-scavenging and anti-inflammatory effects (Fig. 8a). This study indicates that optimizing particle size alone can achieve clinically meaningful organ-specific delivery. This principle of size-dependent accumulation is also applicable to other organs with distinct vascular architectures. For instance, in the treatment of acute kidney injury (AKI), Han et al*.* [230] designed mesoscale PDA nanoparticles with a diameter of approximately 400 nm (MeNP₄ᵀᴾ) loaded with triptolide to achieve specific targeting of renal tubular cells. The unique glomerular filtration apparatus of the kidney establishes a size-dependent retention threshold where particles smaller than roughly 10 nm undergo rapid renal clearance, whereas particles in the mesoscale range of approximately 50 to 500 nm can selectively lodge within the renal tubules. By tuning the size of PDA nanoparticles to approximately 400 nm, this system achieved preferential accumulation in damaged kidney tissues to specifically deliver triptolide to tubular cells while minimizing systemic exposure. Combined with the intrinsic antioxidant activity of PDA, this size-optimized platform significantly alleviated oxidative stress and suppressed inflammatory cell activity while promoting the generation of regulatory T cells in a cisplatin-induced AKI model.

These examples reveal two complementary principles for achieving size-dependent passive accumulation with PDA-based nanoplatforms where the first dictates that different organs possess characteristic optimal size thresholds for accumulation such as approximately 680 nm for lung targeting and 400 nm for kidney targeting reflecting their distinct vascular structures and filtration mechanisms. The second principle demonstrates that organ-specific delivery can be achieved solely through size optimization without complex biological modifications to simplify the formulation while preserving targeting efficacy. By capitalizing on the highly tunable synthesis of PDA nanoparticles, researchers can exploit these physiological size filters to achieve passive yet selective accumulation at disease sites.

#### Mucoadhesive Retention

Mucosal surfaces including the ocular and gastrointestinal mucosa serve as both barriers against pathogen invasion and targets for local drug delivery. However, rapid mucus turnover, mucociliary clearance, and enzymatic degradation frequently limit drug retention at these sites and necessitate frequent administration to maintain therapeutic concentrations [231]. The catechol groups of PDA form hydrogen bonds and covalent interactions with mucin glycoproteins while its amine groups participate in electrostatic interactions with negatively charged mucus components alongside its film-forming ability that enables it to uniformly coat mucosal surfaces. These characteristics render PDA an ideal platform for prolonging local drug retention across various mucosal tissues.

A representative example arises in ocular surface diseases. In the treatment of dry eye disease, Yang et al*.* [55] developed PBA-modified PDA nanoparticles loaded with melatonin (PPP@MT) formulated as eye drops. To prolong ocular surface retention, the formulation incorporated poly-vinyl alcohol, which increased viscosity and extended the residence time of the nanoparticles. This enhanced bioavailability enabled sustained ROS scavenging in corneal tissues. The system achieved synergistic therapeutic effects through prolonged antioxidant activity and the downregulation of the NLRP3/NF-κB/IL-17 inflammatory pathways to demonstrate that mucoadhesive retention can transform conventional eye drops into long-acting topical therapeutics. Beyond ocular applications, this strategy has also been utilized for gastrointestinal mucosal delivery. In the treatment of IBD Guan et al*.* [106] developed PAA-coated MPDA nanoparticles (PAA@MPDA-SAP) for oral administration. The pH-responsive behavior of the PAA coating protected the nanoparticles from gastric degradation and enabled specific drug release at the inflamed colon while its carboxyl groups enhanced mucoadhesion through electrostatic interactions with intestinal mucus. Furthermore, the negatively charged surface facilitated preferential accumulation in inflamed colonic tissues to achieve site-specific retention within the gastrointestinal tract. In addition to open mucosal surfaces, the adhesive properties of PDA can significantly prolong the local action time of drugs within enclosed pathological cavities subjected to continuous fluid clearance mechanisms such as the articular cavity. For the treatment of OA, Zong et al*.* [232] developed KGN-loaded PLGA/PDA core–shell nanoparticles (KGN@PLGA/PDA-PEG-E7) for intra-articular injection. In OA, the rapid turnover of synovial fluid typically leads to the swift clearance of injected drugs, but this system leveraged the intrinsic tissue-adhesive capability of the PDA shell combined with PEGylation and E7 peptide modification to effectively overcome the synovial clearance barrier and substantially prolong the retention time of the nanoparticles on the damaged cartilage surface. This PDA-based in situ anchoring effect created a long-term stable biochemical microenvironment within the joint cavity for the pH-responsive sustained release of KGN to thereby support continuous cartilage regeneration.

Beyond cavities and mucosal surfaces, the adhesive properties of PDA can also be utilized to achieve robust integration between implant materials and solid tissues. Yang et al*.* [233] developed PDA nanoparticle-decorated stem cell spheroid-derived decellularized ECM (PDANP-decorated 3D dECM) for traumatic brain injury (TBI) repair. Unmodified 3D dECM lacks tissue adhesiveness and easily detaches from the injury site post-implantation to necessitate fixation with fibrin glue. Following PDA modification multiple 3D dECM units can self-assemble into larger structures via the catechol groups of PDA and directly adhere to the moist brain tissue surface while maintaining attachment even after PBS flushing. This tissue-adhesive capability allows the 3D dECM to remain stably anchored at the lesion site to continuously exert its antioxidant and neuroprotective effects (Fig. 8b). This study expanded the application of the adhesive properties of PDA from mucosal surfaces to solid tissues to demonstrate its immense potential in the fixation of tissue engineering scaffolds.

The aforementioned examples illustrate that by leveraging the versatile surface chemistry of PDA, mucoadhesive retention can be customized according to the distinct physiological characteristics of various mucosal surfaces to transform conventional dosage forms into long-acting local therapeutics. Concurrently on solid tissue surfaces the direct adhesion capability of PDA ensures the stable fixation of implanted materials. This broad applicability ranging from nanoparticles to macroscopic scaffolds and from mucosal surfaces to solid tissues establishes PDA as a powerful tool for realizing long-acting localized therapies.

#### pH/NIR/ROS-Responsive Release

Stimuli-responsive drug delivery systems can specifically release therapeutic payloads at disease sites to serve as a powerful strategy for enhancing efficacy while minimizing off-target effects [234–236]. Due to its inherent sensitivity to various pathological signals, PDA has emerged as an ideal platform for stimuli-responsive delivery across multiple diseases [237] to thereby provide precise spatiotemporal control for complex delivery networks.

First, utilizing the characteristic acidic microenvironment of diseased tissues to achieve pH-responsive drug release is one of the most intuitive and widely applied design strategies. Ain et al*.* [238] constructed aminated mesoporous silica nanoparticles loaded with Glycyrrhiza polysaccharide and further coated them with a PDA layer (MSN-NH_2_-GGP@PDA) for the topical treatment of acne. Utilizing the pH-responsive nature of PDA, this platform achieved a cumulative release rate of up to 89% under simulated acidic acne skin conditions, which was significantly higher than that in a neutral environment. This study validated the feasibility of PDA as a pH-responsive functional coating to demonstrate the application potential of PDA-based pH-responsive drug delivery systems in dermatological diseases. Beyond pH responsiveness, the photothermal properties of PDA enable on-demand release triggered by NIR irradiation. Chen et al*.* [239] prepared alginate microcarriers via microfluidic electrospray technology and surface-modified them with PDA nanoparticles to construct an NIR-responsive drug delivery system (PDA@microcarriers-ALA). Capitalizing on the excellent photothermal conversion capability of PDA the system achieved a localized temperature increase to approximately 40 °C under NIR irradiation to thereby trigger the precise release of loaded α-lipoic acid and effectively alleviate hair cell damage and hearing loss (Fig. 8c). This research expanded the application of PDA-responsive systems to the treatment of sensorineural hearing loss while showcasing a material design strategy that combines microfluidic technology with PDA modification.

Another effective strategy utilizes the susceptibility of PDA to oxidative degradation to achieve ROS-responsive release. Gao et al*.* [240] developed a PDA-coated black phosphorus nanocarrier (BP@P + MP) loaded with methylprednisolone for the treatment of ALI. The PDA shell endowed the nanocarrier with ROS responsiveness to degrade and release the drug within the ROS-rich inflammatory microenvironment. The system also exhibited excellent ROS-scavenging capabilities to effectively reduce intracellular ROS levels through the synergistic action of BP and PDA (Fig. 8d). This study extended the application of PDA-responsive systems to acute inflammatory diseases and demonstrated a design strategy for black phosphorus-PDA composites. Beyond the aforementioned single responsiveness, PDA can concurrently respond to multiple pathological stimuli to achieve multi-responsive release. Da et al*.* [241] constructed a PDA-coated hollow mesoporous Prussian blue nanoplatform loaded with curcumin (HMPB@Cur@PDA) for the treatment of maxillofacial infections. Within this system PDA acted as a responsive gating coating capable of simultaneously reacting to the acidic conditions at pH 5.0 and the excessive ROS of the inflammatory microenvironment. In vitro release studies showed that the cumulative release rate reached 44.5% at 60 h under pH 5.0 conditions, whereas the release rate further increased to 55.1% after the addition of H_2_O_2_ to simulate a ROS environment which was significantly higher than the 21.1% observed under neutral conditions. This dual pH/ROS responsiveness fully exploited the chemical properties of PDA to enable more precise drug release within the inflammatory microenvironment.

These examples reveal three distinct mechanisms of PDA-based stimuli-responsive release that include utilizing protonation-induced structural loosening within acidic microenvironments to achieve pH-responsive release, disrupting drug–pore interactions via photothermal effects for NIR-triggered release, and employing the oxidative degradation of the PDA shell for ROS-responsive release. By combining these responsive behaviors with the intrinsic therapeutic functions of PDA, these platforms achieve precise spatiotemporal control over drug delivery to enhance efficacy while minimizing off-target effects.

#### Environmental Buffering: PDA as an Interfacial Protective Layer

Distinct from the aforementioned therapeutic strategies relying on drug delivery or signal regulation, the unique physicochemical attributes of the PDA material itself enable it to serve as an active interfacial protective layer under conditions of environmental exposure. As a biomimetic analog of melanin, PDA possesses an extended π–π-conjugated structure and exhibits broad-spectrum light absorption characteristics across the UV–Vis–NIR bands while it can convert photonic energy into thermal or vibrational energy through non-radiative energy dissipation to thereby attenuate incident ultraviolet radiation at the material–tissue interface [24]. This energy-quenching capability based on the intrinsic optical properties of the material enables PDA to reduce the initial photonic stress before the amplification of biological signaling cascades.

For example, Zhang et al*.* [242] reported that melanin-like PDA nanoparticles significantly alleviated epidermal thickening and oxidative DNA damage in an ultraviolet B-induced mouse skin injury model. Studies indicate that this protective effect primarily originates from the broad-spectrum ultraviolet absorption capability and ROS quenching properties of PDA rather than signal regulation mediated by additional drug components. Experimental results showed that the ultraviolet-induced ROS levels in the skin of the PDA-treated group were significantly reduced and the epidermal structural integrity was better maintained to validate its potential as a direct photoprotective material. In addition to radiation shielding PDA can exert a continuous redox buffering function at the air-skin interface. The catechol/quinone redox pairs within its structure enable it to continuously scavenge ROS generated by exposure to ultraviolet radiation, air pollutants, or particulate matter. Sunoqrot et al*.* [243] demonstrated that the deposition of melanin-like PDA nanoparticles with tuned surface chemistry in ex vivo human skin was significantly enhanced owing to the strong adhesion between catechol groups and stratum corneum proteins. The enhanced skin retention capability enabled PDA to maintain a more durable ROS-scavenging effect under simulated environmental stress conditions to emphasize the importance of interfacial adhesiveness for long-term protective performance. Furthermore, PDA has been integrated into surface coatings or hydrogel systems to construct topical barrier materials with environmental buffering functions. Yazdi et al*.* [244] summarized in a review that various PDA-based skin materials exhibited enhanced structural stability, moisturizing performance, and antioxidant tolerance when utilized as topical films or dressings. Notably this oxidative buffering function can be maintained without relying on additional active drugs to further reflect the intrinsic redox regulatory capability of PDA as a functional material.

In summary, PDA should not merely be regarded as a drug delivery carrier but should be redefined as an active protective material with tunable structure, strong interfacial adhesiveness, and continuous oxidative buffering capacity. Its broad-spectrum light absorption, energy dissipation, and surface-anchoring characteristics endow it with unique advantages in tissues subjected to long-term environmental exposure.

## Clinical Translation and Pharmacokinetic Challenges

As described above, PDA-based nanoplatforms have demonstrated exceptional intervention capabilities across multiple dimensions such as breaking the ROS-inflammation feedback loop, overcoming physiological delivery barriers, remodeling regenerative microenvironments, and regulating programmed cell death. However, although PDA-based nanoplatforms exhibit significant therapeutic potential in various oxidative stress-related disease models, their translation from the laboratory into clinical application still faces numerous translational bottlenecks. To accurately evaluate the clinical value of PDA-based materials in cutting-edge fields such as antioxidant nanozymes, melanin-like materials, and ROS-regulating nanotherapies, future research should shift from singular efficacy validation to systematic pharmacokinetic analysis and the evaluation of clinical translational parameters [245].

### Pharmacokinetics and Long-Term In Vivo Fate

Currently, most research relies on short-term imaging techniques to evaluate the in vivo distribution of PDA and lacks systematic quantitative pharmacokinetic data such as half-life, area under the curve, and systemic clearance. As exogenous nanomaterials, intravenously injected PDA nanoparticles are rapidly taken up by the mononuclear phagocyte system and typically accumulate heavily in the liver and spleen, which is consistent with the in vivo fate of the vast majority of nanomedicines [246]. Although PDA possesses melanin-like biodegradability and can undergo slow degradation under high concentrations of ROS or specific enzymatic actions [247], the complete clearance pathway of PDA such as whether it occurs via renal or hepatobiliary excretion and the exact degradation kinetics remain incompletely elucidated. For instance, surface modifications such as PEGylation [93] can effectively prolong its blood half-life and reduce non-specific protein adsorption, but they may simultaneously delay its ultimate biodegradation and excretion processes. Recent studies have also demonstrated that particle size significantly influences the toxicity profile of PDA nanoparticles, with approximately 100—nm-sized particles exhibiting lower toxicity compared to both smaller (50 nm) and larger (200 nm) counterparts [248]. Future research should integrate precise tracing technologies such as isotope labeling to clarify the long-term in vivo fate of PDA and its degradation products to evaluate potential accumulation and toxicity risks in target organs.

### Route of Administration Optimization and Dosage Considerations

The choice of administration route directly impacts the bioavailability and systemic safety of PDA nanoplatforms. Intravenous injection enables systemic delivery but inevitably faces issues such as serum protein adsorption to form a protein corona and premature clearance by the immune system within the complex blood environment [249]. In contrast, optimizing local administration strategies serves as an effective approach to accelerate the clinical translation of PDA. Examples include intra-articular injection for the treatment of OA, oral hydrogel delivery for IBD, and topical dressings for wound healing [182, 202, 232]. Local administration can not only maximally bypass the biological barriers of systemic blood circulation to increase local drug exposure at the lesion but also significantly reduce off-target effects and systemic toxicity. Furthermore, determining the minimum effective dose and maximum tolerated dose while exploring the administration frequency under different delivery routes represents a core component of future preclinical research.

### Long-Term Biosafety and Toxicological Evaluation

Although in vitro cell experiments and short-term mouse model studies have confirmed the excellent biocompatibility of PDA, achieving ultimate clinical translation still requires the support of long-term toxicological data. As previously mentioned, high concentrations of PDA may exert certain impacts on blood coagulation and complement system activation [61]. However, existing biosafety evaluations are mostly limited to mouse models and future toxicological assessments complying with good laboratory practices and regulatory guidelines should be conducted in large animal models [250] to particularly investigate whether long-term exposure to PDA induces immunogenicity, reproductive toxicity, or genotoxicity. Notably, it is also necessary to investigate whether PDA degradation products such as dopamine derivatives [60] can cross the BBB or accumulate within specific organs to thereby trigger irreversible side effects in the nervous or cardiovascular systems. Furthermore, the intrinsic redox activity of PDA is highly dependent on microenvironmental conditions. As systematically elucidated by Liu et al*.* [26], although PDA is primarily and widely utilized as a ROS scavenger, it can also exhibit anomalous pro-oxidant behavior under specific conditions. For example, under highly reduced states, the catechol groups within the PDA structure can transfer electrons to dissolved oxygen in the environment to thereby generate ROS. Although this pro-oxidant property can be ingeniously applied in specific therapeutic areas such as antibacterial treatments, it poses a potential toxicity risk to normal tissues. Therefore, precisely understanding and regulating the in vivo redox state of PDA to prevent off-target oxidative stress damage to healthy tissues remains a critical consideration in its safe progression toward clinical application.

### Manufacturing Feasibility

Developing a PDA-based nanoplatform capable of standardized large-scale preparation is crucial for advancing its clinical translation in the treatment of oxidative stress-related diseases. Currently, the synthesis of PDA nanoparticles largely relies on the self-oxidation polymerization of dopamine under alkaline conditions. This process is influenced by multiple factors such as pH, temperature, oxidant type, and reaction time. Thus, even minor process variations can lead to significant differences in particle size, morphology, and surface chemistry, which in turn affect its antioxidant performance and biodistribution behavior. Furthermore, complex steps such as template methods, metal doping, and surface functionalization are often introduced to enhance therapeutic efficacy. Although this synthetic approach is relatively controllable at the laboratory scale, scaling it up to industrial-scale production further increases process difficulty and the risk of batch-to-batch variations, disadvantaging quality control and compliant manufacturing. Therefore, future research should dedicate efforts to developing simplified and robust synthetic strategies that optimize reaction conditions and purification processes to thereby facilitate the smooth transition of PDA-based nanoplatforms from the laboratory to clinical application.

## Conclusion and Outlook

With the rapid development of nanobiomaterials, PDA-based nanoplatforms have demonstrated immense translational potential in intervening in oxidative stress-mediated diseases. This review systematically summarizes the unique physicochemical properties of PDA alongside its antioxidant mechanisms such as ROS-scavenging and enzyme-mimicking activities, and its structural engineering strategies and diverse morphologies in constructing delivery systems. Building upon this foundation, we highlight the multidimensional framework of PDA in the intervention of oxidative stress diseases. Specifically, we systematically summarize its core strategic roles in breaking the self-amplifying ROS-inflammation loop, overcoming complex biological barriers, remodeling the regenerative microenvironment, regulating programmed cell death cascades, and achieving multimodal synergistic therapy. These synergistic strategies significantly broaden the boundaries of antioxidant interventions. By systematically analyzing the inherent biocompatibility and stimuli-responsive behavior of PDA alongside its dual function as both a structural carrier and an active redox regulator, this article evaluates its practical feasibility and therapeutic efficacy as a nano-antioxidant to provide a solid theoretical basis and design paradigm for the future development of next-generation PDA-based nanoplatforms featuring high targeting capability and deep synergistic therapeutic functions. However, despite these advancements, the transition from fundamental research to clinical application still requires resolving several conceptual and methodological limitations. Future research should prioritize the following four dimensions to enhance the therapeutic precision and translational potential of PDA-based nano-antioxidants.

1. Enhancing catalytic efficiency through single-atom engineering. The inherent kinetics of ROS scavenging by PDA are often insufficient to address the characteristic acute oxidative bursts in diseases such as sepsis or ischemia–reperfusion injury, where integrating single-atom catalysis (SAC) represents a frontier direction for overcoming this kinetic bottleneck [251]. By utilizing the robust metal-chelating capability of PDA to anchor transition metal single atoms such as Fe, Cu, or Mn within its polymer backbone researchers can promote strong metal-support interactions. Indeed, recent pioneering studies have demonstrated that anchoring isolated transition metal atoms onto a melanin-like network can achieve orders-of-magnitude amplification in nanozyme or antioxidant activities [84]. This strategy not only maximizes atomic utilization but also significantly reduces the activation energy for ROS degradation to transform PDA from a stoichiometric scavenger into a highly efficient catalytic platform for selective ROS clearance.
2. Addressing the ROS paradox through stimuli-responsive systems. A key challenge in antioxidant therapy is the ROS paradox where maintaining physiological levels of ROS is crucial for essential signal transduction and immune functions. Indiscriminate and long-term clearance may inadvertently lead to antioxidant stress and disrupt homeostatic balance. Therefore, future designs must shift toward intelligent and closed-loop regulators where the introduction of ROS-responsive chemical groups can ensure that antioxidant activity is strictly confined spatially and temporally within hyperoxidative niches [201, 252]. Furthermore, utilizing the intrinsic imaging modalities of PDA such as photoacoustic or MRI can enable the development of theranostic systems with real-time monitoring and on-demand intervention capabilities to thereby facilitate the formulation of personalized treatment regimens [253, 254].
3. Elucidating mechanisms through multi-omics. Currently, the evaluation of PDA therapeutic efficacy typically relies on endpoint phenotypic observations where the underlying molecular interactions remain largely unexplored. To systematically understand how PDA reshapes the pathological landscape, the application of multi-omics technologies such as single-cell RNA sequencing and spatial transcriptomics is crucial [255, 256]. Recent breakthroughs applying single-cell sequencing in nanomedicine have revealed [257] that nanotherapeutics can induce profound transcriptomic alterations within specific immune cell subsets at a resolution unattainable by traditional bulk-tissue analysis. These methodologies can elucidate the impact of PDA on organelle crosstalk and its potential roles in regulating systemic axes such as the microbiome-immune-organ axis where these insights hold profound significance for identifying off-target effects and ensuring long-term biosafety.
4. Rational design assisted by computational chemistry and artificial intelligence. The complexity and stochasticity of the dopamine polymerization process remain major obstacles to standardizing the production of disease-specific materials. Moving away from traditional trial-and-error approaches, integrating artificial intelligence and density functional theory calculations provides a pathway for rational design. Computational simulations of electron-transfer energy barriers and conformational evolution at the sub-nanometer scale facilitate the establishment of quantitative structure–activity relationship models. As demonstrated by recent predictive frameworks for other nanomaterials [258, 259], this integration of AI and machine learning will enable the predictive optimization of physicochemical parameters such as particle size and surface charge to thereby accelerate the development of customized next-generation PDA-based nanoplatforms.

In conclusion, although the clinical translation of PDA-based nano-antioxidants still faces significant challenges, their tunable melanin-like chemical properties and proven biocompatibility provide a solid foundation for further development. Overcoming current bottlenecks requires moving away from empirical formulation explorations to integrate single-atom engineering, responsive biomaterials, multi-omics analysis, and computational modeling. By embracing these interdisciplinary approaches, the next generation of PDA-based nanoplatforms holds the promise to transcend the limitations of current antioxidants, ultimately translating the bioinspired wisdom of melanin into tangible clinical benefits for patients suffering from oxidative stress-mediated diseases.

## Abbreviations

AD: Alzheimer's disease
AKI: Acute kidney injury
ALI: Acute lung injury
BBB: Blood–brain barrier
CAT: Catalase
cfDNA: Circulating free DNA
CQDs: Carbon quantum dots
DFO: Deferoxamine
DHI: 5,6-Dihydroxyindole
ECM: Extracellular matrix
EGCG: Epigallocatechin gallate
ET: Electron transfer
ETC: Electron transport chain
GPx: Glutathione peroxidase
GPX4: Glutathione peroxidase 4
H_2_O_2_: Hydrogen peroxide
HA: Hyaluronic acid
HAT: Hydrogen atom transfer
HO_2_: Hydroperoxyl radicals
HPDA: Hollow PDA
IBD: Inflammatory bowel disease
KGN: Kartogenin
LF: Liver fibrosis
MM: Macrophage membrane
MOF: Metal–organic framework
MPDA: Mesoporous PDA
MRI: Magnetic resonance imaging
NIR: Near-infrared
O_2_^·^^−^: Superoxide anion radicals
OA: Osteoarthritis
OXPHOS: Oxidative phosphorylation
PAA: Polyacrylic acid
PBA: Boronophenylalanine
PDA: Polydopamine
NDs: Nanodots
PEG: Polyethylene glycol
POD: Peroxidase
RA: Rheumatoid arthritis
RBC: Red blood cell
ROS: Reactive oxygen species
SCI: Spinal cord injury
SOD: Superoxide dismutase
TBI: Traumatic brain injury
UC: Ulcerative colitis
